# Ultrathin Monatomic Antimony Films by Sacrificial Atomic Layer Deposition for Phase Change Memory

**DOI:** 10.1002/adma.202519924

**Published:** 2025-11-29

**Authors:** Gwangsik Jeon, Sangmin Jeon, Seunghwan Lee, Jeong Woo Jeon, Wonho Choi, Byongwoo Park, Sungjin Kim, Chanyoung Yoo, Hyejin Jang, Cheol Seong Hwang

**Affiliations:** ^1^ Department of Materials Science and Engineering, and Inter‐University Semiconductor Research Center Seoul National University Seoul 08826 Republic of Korea; ^2^ Department of Materials Science and Engineering Seoul National University Seoul 08826 Republic of Korea; ^3^ Department of Materials Science and Engineering Hongik University Seoul 04066 Republic of Korea

**Keywords:** antimony, atomic layer deposition, local epitaxial growth, nanoscale electronics, phase change memory

## Abstract

Antimony (Sb) is an intriguing material for advanced electronics, with thickness‐dependent properties at the nanoscale offering new functionalities. However, conventional methods for depositing Sb thin films cannot produce continuous ultrathin films with conformality in complex nanoscale structures. This study introduces a novel sacrificial atomic layer deposition (s‐ALD) approach that overcomes these limitations by using chemical substitution between the antimony precursor and the pre‐deposited Sb_2_Te_3_. The structural similarity between Sb_2_Te_3_ and Sb enables local epitaxial growth of a uniform, (00*l*)‐oriented Sb film with exceptional surface smoothness (root‐mean‐squared roughness << 1 nm) at a 4‐nm thickness. Highly pure Sb films with excellent wafer‐scale uniformity and conformality are achieved on high‐aspect‐ratio structures. The mechanism involves substitution reactions driven by the preferential Te‐(CH_3_)_3_Si bonding, along with enhanced atomic diffusion through the aligned crystal structure. Phase change memory devices using 5‐nm‐thick s‐ALD Sb films demonstrate ultrafast switching with femtosecond laser pulses (≈220 fs) with high device‐to‐device uniformity (coefficient of variation < 5 %) and ultralow drift coefficients (0.0013 for the on state and 0.0073 for the off state). This s‐ALD technique offers a promising pathway for depositing ultrathin, uniform Sb films, enabling full utilization of Sb's unique nanoscale properties.

## Graphical abstract

A three‐step sacrificial atomic layer deposition (s‐ALD) process enables wafer‐scale growth of ultrathin and uniform antimony (Sb) thin films. An ultrathin (≈4 nm) Sb film is uniformly deposited with its characteristic bilayer structure (Sb‐Sb) aligned along the substrate. The excellent surface coverage of the s‐ALD allows facile electrical conduction even in the few‐nanometer thickness range.

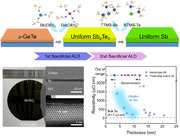

## 1 Introduction

Antimony (Sb) is an intriguing semimetal with high carrier mobility, low thermal conductivity, and a unique electronic structure,^[^ ^1^, ^2^, ^3^, ^4^ ^]^ which enables low‐resistance source and drain contacts with a 2D semiconductor, such as MoS_2_, channel in advanced transistor technologies.^[^ ^3^ ^]^ Moreover, when the Sb film thickness is a few nanometers, the stability of the amorphous state increases, enabling it to serve as a monoatomic phase change memory (PCM) material.^[^ ^4^, ^5^, ^6^ ^]^ Using pure Sb in PCM may overcome issues linked to complex materials, such as compositional partitioning and resistance drift during operation.^[^ ^4^, ^5^ ^]^ Furthermore, the programmable optical nonlinearities of Sb thin films offer great potential for nanophotonic and optoelectronic applications.^[^ ^7^, ^8^ ^]^ Previous studies on device implementation using Sb thin films have mainly relied on physical vapor deposition methods, which face challenges with conformal deposition over complex 3D structures. Conformality on high‐aspect‐ratio (HAR) structures is particularly important in PCM because it enables cost‐effective, high‐density integration by greatly reducing the number of lithography steps required.^[^ ^9^, ^10^ ^]^ Atomic layer deposition (ALD) offers the most promising approach for conformally depositing ultrathin Sb films. However, previous works on Sb ALD have been suboptimal, especially when trying to grow ultra‐thin films.^[^ ^11^, ^12^, ^13^ ^]^ The main challenge comes from the island growth mechanism during the early stages of film deposition.^[^ ^14^ ^]^ Furthermore, polycrystalline films formed through island growth result in significant surface roughness and non‐uniformity, which can severely affect device performance and reliability.^[^ ^15^ ^]^ Therefore, a fundamentally different approach is needed to grow ultrathin and uniform Sb films.

This study introduces a new chemical film growth method for creating ultrathin, continuous Sb films using sacrificial ALD (s‐ALD), utilizing the previously reported uniform polycrystalline two‐dimensional (2D) Sb_2_Te_3_ film with its c‐axis preferably aligned to the out‐of‐plane direction.^[^ ^16^ ^]^ Sb has a 2D crystal structure with a suitable lattice match to Sb_2_Te_3_, suggesting that the well‐aligned Sb_2_Te_3_ can serve as a template for uniformly growing c‐axis aligned ultrathin Sb films (<5 nm). The process involves chemical substitution reactions between metal‐organic precursors and pre‐deposited films, starting with an amorphous GeTe (*a*‐GeTe) buffer layer, which is then converted to crystalline Sb_2_Te_3_ (*c*‐Sb_2_Te_3_) and ultimately to monatomic crystalline Sb (*c*‐Sb), maintaining a smooth morphology and free of detectable impurities. Additionally, phase change memory devices based on ultrathin Sb films were fabricated, and their switching performance was tested. The devices showed reliable, ultrafast reversible phase change upon femtosecond laser irradiation, with a high on/off ratio, low device‐to‐device variation, and an extremely low resistance drift coefficient. This method offers a promising way to overcome the limitations of conventional Sb deposition techniques, enabling full utilization of Sb's unique properties in the ultrathin regime while ensuring film uniformity and conformality.

## 2 Growth of an Ultrathin Monatomic Sb Film by s‐ALD

**Figure 1:**
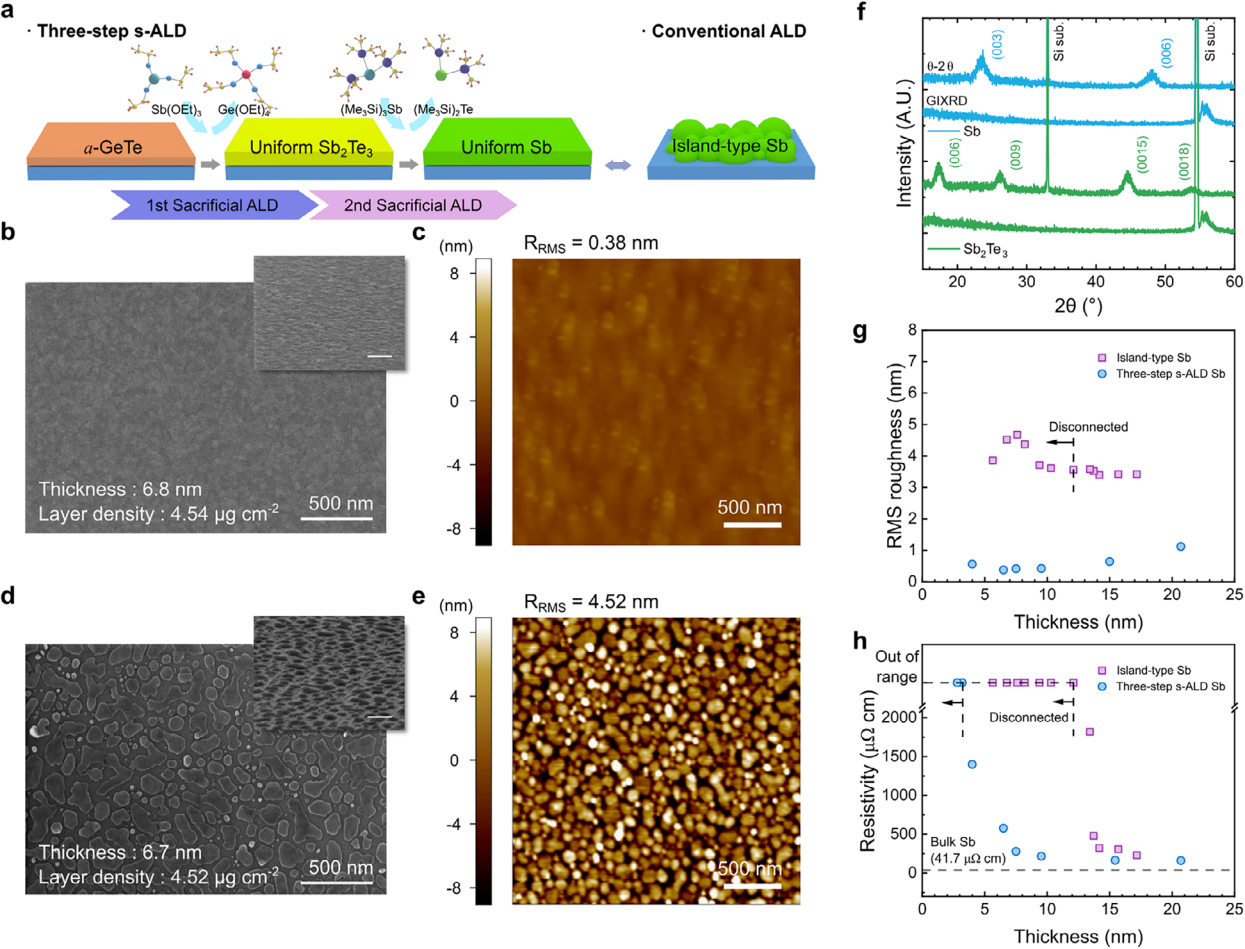
Ultrathin *c*‐Sb film growth via s‐ALD. a) Schematic illustration of the three‐step s‐ALD process for uniform Sb growth compared to rough Sb from conventional ALD. b, c) SEM (b) and AFM (c) images of 6.8‐nm‐thick *c*‐Sb film grown by the three‐step s‐ALD. d,e) SEM (d) and AFM (e) images of 6.7‐nm‐thick island‐type *c*‐Sb. Inset scale bar: 500 nm. f) X‐ray diffractogram of the *c*‐Sb_2_Te_3_ and *c*‐Sb films grown by the three‐step s‐ALD. g, h) RMS roughness (g) and resistivity (h) as functions of film thickness for the three‐step s‐ALD *c*‐Sb and the island‐type *c*‐Sb.

A three‐step s‐ALD process is used to deposit the ultrathin monatomic Sb film (**Figure** **1**a). The first step involves depositing an *a*‐GeTe sacrificial buffer layer through the sequential injection and purging of the Ge precursor (Ge^II^NMe_2_[(N*i*Pr)_2_CNMe_2_], where Me = CH_3_, *i*Pr = (CH_3_)_2_CH‐) and the Te precursor ((Me_3_Si)_2_Te) at 130 °C. NH_3_ is used as a reaction‐enhancing gas and is co‐injected with the Te precursor.^[^ ^17^ ^]^ Next, *c*‐Sb_2_Te_3_ is deposited on top of the *a*‐GeTe buffer layer as the second step by sequential injection and purging of the Sb precursor (Sb(OEt)_3_, where Et = C_2_H_5_) and the Te precursor at 170 °C, again with NH_3_ co‐injection.^[^ ^18^ ^]^ During this step, the Ge in the underlying *a*‐GeTe buffer film is chemically substituted with Sb because of the favorable bonding between ‐OEt and Ge, which causes the formation of volatile Ge(OEt)_4_. This active chemical substitution results in a *c*‐Sb_2_Te_3_ film with a highly uniform morphology, unlike the rough surfaces typical of conventional island‐type ALD. Ultimately, the initial *a*‐GeTe buffer layer completely transforms into a c‐axis aligned, *c*‐Sb_2_Te_3_ layer.^[^ ^16^ ^]^ To produce monatomic *c*‐Sb films in the third step, (Me_3_Si)_3_Sb is pulsed onto the aligned *c*‐Sb_2_Te_3_ film at 220 °C. The stronger bonding of Me_3_Si‐ with Te than with Sb drives the substitution of Te in the *c*‐Sb_2_Te_3_ film with Sb during this process. Repeating the pulse and purging of the (Me_3_Si)_3_Sb precursor eventually replaces all Te atoms within the *c*‐Sb_2_Te_3_ film with Sb, resulting in a uniform, aligned, monatomic *c*‐Sb film. Section S1 (Supporting Information) with Figure S1 (Supporting Information) provides more detailed experimental and theoretical information about the three‐step s‐ALD process.

Figure 1b and c show scanning electron microscopy (SEM) and atomic force microscopy (AFM) images of the surface morphology of a 6.8‐nm‐thick *c*‐Sb film deposited by the three‐step s‐ALD on the SiO_2_ substrate. For comparison, a 6.7‐nm‐thick *c*‐Sb film was deposited by a two‐step ALD process (skipping the *a*‐GeTe growth step from the three‐step s‐ALD), which shows typical island‐type surface morphology (Figure 1d, e). Figure S2 (Supporting Information) compares the morphology evolution of each process. It is noted that the attempts to grow Sb films using the direct reaction between Sb(OEt)_3_ and (Me_3_Si)_3_Sb^[^ ^13^ ^]^ were unsuccessful, which may be due to the unfavorable reactor design.^[^ ^19^, ^20^ ^]^ Consequently, only the *c*‐Sb film deposited via the three‐step s‐ALD approach exhibited a smooth morphology with a low root‐mean‐squared roughness (R_RMS_) value of 0.38 nm (Figure 1c) even at such a thin thickness. In contrast, the *c*‐Sb film via the two‐step ALD exhibits a rough surface (R_RMS_ of 4.52 nm), characterized by an island‐type morphology with incomplete substrate coverage (Figure 1d, e). Figure S3 (Supporting Information) shows AFM line scans of both three‐step s‐ALD and island‐type *c*‐Sb films, illustrating the clear morphological difference.

Figure 1f shows the θ‐2θ X‐ray diffraction (XRD) and grazing‐incidence XRD (GIXRD) results of the *c*‐Sb_2_Te_3_ and *c*‐Sb films grown by the three‐step s‐ALD. Before the *c*‐Sb film growth, the peaks corresponding to Sb_2_Te_3_ (006), (009), (0015), and (0018) planes are observed in the θ‐2θ XRD pattern, and no peaks appear in the GIXRD pattern, indicating that its c‐axis is aligned along the out‐of‐plane direction. After the third step, only the Sb (003) and (006) planes are observed in the θ‐2θ XRD pattern, and no peaks are observed in the GIXRD pattern. These findings indicate that the <00*l*> directions of polycrystalline Sb and the Sb_2_Te_3_ films are aligned to the out‐of‐plane direction. It is worth noting that Sb_2_Te_3_ and Sb both belong to the same *R3̅m* space group with a low in‐plane lattice constant mismatch of ≈1 % (Sb_2_Te_3_ a = 4.26 Å, Sb a = 4.31 Å, Figure S4, Supporting Information).^[^ ^21^, ^22^ ^]^ Such crystallographic similarity allows the local epitaxial growth of Sb on the aligned Sb_2_Te_3_ so that the resulting monatomic *c*‐Sb film is (00*l*)‐aligned with smooth film morphology.^[^ ^23^, ^24^ ^]^ Meanwhile, their in‐plane orientations were random as they are polycrystalline materials.

Figure 1g shows R_RMS_ variations as a function of *c*‐Sb film thickness grown by two different methods. The *c*‐Sb films grown using three‐step s‐ALD exhibit excellent morphology, with R_RMS_ below 1 nm for thicknesses under 15 nm. In contrast, the island‐like *c*‐Sb film grown by two‐step s‐ALD shows R_RMS_ exceeding 3 nm across all film thicknesses (Figure S5, Supporting Information, shows AFM images of both types of *c*‐Sb films across the full thickness range, demonstrating the consistent morphological difference). Also, the *c*‐Sb film grown by the three‐step s‐ALD completely covers the substrate, even at a thickness of ≈4 nm (Figure S6, Supporting Information). These differences in morphology lead to a pronounced contrast in electrical conductivity between the two types of films (Figure 1h). For the three‐step method, the electrical conductivity can be measured (indicating a continuous film) down to ≈4 nm thickness, and the film resistivity quickly approaches the bulk Sb resistivity at thicknesses above ≈10 nm.^[^ ^30^ ^]^ Conversely, the island‐type films do not exhibit reliable electrical conductance below ≈13 nm, indicating discontinuity. Furthermore, the Hall measurement of the 6.5‐nm‐thick *c*‐Sb film deposited by three‐step s‐ALD revealed a resistivity of 537.8 µΩ cm and a mobility of 111 cm^2^ V^−1^ s^−1^. Notably, this mobility exceeds that of typical polycrystalline Sb films (30–50 cm^2^ V^−1^ s^−1^).^[^ ^13^ ^]^ It approaches the mobility of single‐crystalline, few‐layer Sb flakes (≈150 cm^2^ V^−1^ s^−1^),^[^ ^29^ ^]^ demonstrating the benefit of the c‐axis‐aligned film structure. **Table** **1** compares the minimum continuous film thicknesses and electrical properties of Sb films deposited by various methods reported in the literature.^[^ ^11^, ^12^, ^13^, ^25^, ^26^, ^27^, ^28^, ^29^ ^]^ To date, depositing continuous Sb films at a large scale with thicknesses below several nanometers remains challenging. It is noted that direct comparison with literature is limited by the lack of equivalent ultrathin Sb data (< 10 nm), as most studies characterize thicker films. Therefore, the analysis highlights general trends while emphasizing that the continuity of s‐ALD films uniquely enables reliable electrical measurements in this ultrathin regime. This study demonstrates that the three‐step s‐ALD can feasibly enable the deposition of ultrathin (≈4 nm), smooth, and continuous *c*‐Sb films, overcoming the fundamental limitations of conventional deposition methods. In a broader context, while state‐of‐the‐art ALD of metals such as W, Ru, and Co has demonstrated decent electrical performance, these methods often require plasma assistance or higher temperatures and experience island growth during the early stages. In contrast, s‐ALD Sb achieves continuity and reliable electrical performance at temperatures of ≤ 220 °C, making it particularly suitable for depositing ultrathin conformal films with CMOS compatibility.^[^ ^31^, ^32^, ^33^ ^]^

**Table 1:** Benchmarking of Sb Film Deposition Techniques. Comprehensive comparison of film thickness, morphological quality, and electrical properties (resistivity and Hall mobility) for various antimony deposition methods reported in the literature. Note that, as previous reports did not provide quantitative root‐mean‐squared roughness values from AFM measurements, surface roughness was qualitatively evaluated from the available SEM images. (*: Thickness not specified.).

Method	Min. continuous film thickness [nm]	Surface roughness	Resistivity [µΩ cm]	Hall mobility [cm^2 ^V^−1 ^s^−1^]	Refs.
s‐ALD	≈4 nm	Smooth (R_RMS_ = 0.38 nm)	1400.4 at ≈4 nm 537.8 at ≈6.5 nm 161.9 at ∼20.7 nm	111 at ≈6.5 nm	This work
ALD	≈33 nm	Rough	65 at ≈60 nm	Not measured	[11]
ALD	≈6.7 nm	Smooth	98 at ≈36.7 nm	Not measured	[12]
ALD	Not measured	Rough	111.1 at ≈76 nm	76 at ≈76 nm	[13]
ALD	Not measured	Rough	88.5 at ≈69 nm	47 at ≈69 nm	[13]
Thermal evaporation	Not measured	Rough	74*	9*	[25]
Thermal evaporation	≈35 nm	Not Measured	256 at ≈27 nm 59 at ≈230 nm	Not measured	[26]
Thermal evaporation	≈27 nm	Not Measured	177 at ≈35 nm 98 at ≈233 nm	95 at ≈35 nm	[27]
Thermal evaporation	≈1 nm	Smooth	6250 at ≈30 nm	Not measured	[28]
Exfoliation	≈4 nm	Smooth	1200 at ≈4 nm	150 at ≈4 nm	[29]

## 3 Chemical and Structural Characterization

**Figure 2:**
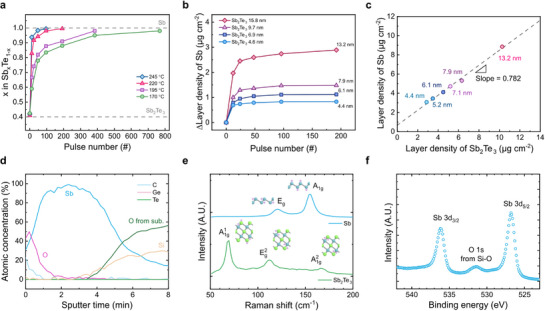
Film Characterization. a) Composition changes of Sb_x_Te_1‐x_ as a function of (Me_3_Si)_3_Sb pulse number in various temperatures measured by XRF. b) Layer density changes of *c*‐Sb films with different initial *c*‐Sb_2_Te_3_ thicknesses as a function of (Me_3_Si)_3_Sb pulse number. c) The relationship between the layer density of the *c*‐Sb_2_Te_3_ buffer layer and the resulting *c*‐Sb film. d) AES depth profile of the *c*‐Sb film. e) Raman spectra of the *c*‐Sb_2_Te_3_ buffer layer and the resulting *c*‐Sb film. f) XPS spectra of the *c*‐Sb film.

**Figure** **2**a shows the film composition (x in Sb_x_Te_1‐x_) measured by X‐ray Fluorescence (XRF) as a function of the number of (Me_3_Si)_3_Sb precursor pulses during the third step at different s‐ALD temperatures. A uniform (00*l*)‐aligned 7‐nm‐thick *c*‐Sb_2_Te_3_ film was selected as a sacrificial buffer layer for all temperatures. The conversion rate from Sb_2_Te_3_ to Sb increases with rising temperature, and the Te in the film is completely removed when the number of pulses is sufficiently increased at all temperatures (768 cycles at 170 °C). Also, the substitution is relatively rapid at the initial pulses, and the conversion process slows down as the number of pulses increases. This indicates that the chemical substitution begins at the film surface and proceeds into the film, where diffusion of elements is necessary for the substitution reaction to proceed.^[^ ^34^ ^]^ As a result, the substitution rate decreases with decreasing temperature due to the slow diffusion of the atoms involved in the substitution reaction. Therefore, subsequent experiments are conducted at 220 °C to achieve facile and complete substitution into Sb within a reasonable number of cycles.

Figure 2b shows the difference in the layer density of Sb during s‐ALD compared to the various initial *c*‐Sb_2_Te_3_ film thicknesses as a function of (Me_3_Si)_3_Sb pulse number at 220 °C. Unlike conventional ALD, where the layer density of the film increases linearly with the number of cycles,^[^ ^35^ ^]^ the *c*‐Sb film thickness self‐saturates according to the initial thickness of the sacrificial *c*‐Sb_2_Te_3_ layer. For *c*‐Sb_2_Te_3_ film thicknesses of 4.6, 6.9, 9.7, and 15.8 nm, the saturated *c*‐Sb thicknesses were 4.4, 6.1, 7.9, and 13.2 nm, respectively, where the film thickness was determined by dividing the layer density by the bulk density of each material. For *c*‐Sb_2_Te_3_, a bulk density of 6.50 g cm^−3^ was used, while for *c*‐Sb films, the bulk density was determined from X‐ray reflectivity (XRR) measurements, which yielded a bulk density of 6.52 g cm^−3^ for the s‐ALD *c*‐Sb films (Figure S7, Supporting Information). Figure 2c illustrates the linear relationship between the layer density of the *c*‐Sb_2_Te_3_ buffer layer (x‐axis) and the resulting *c*‐Sb film (y‐axis), showing the linear dependence with a slope of 0.782 from the best linear fitting. The chemical substitution reaction for the formation of *c*‐Sb can be written as follows:

(1) $${\mathrm{Sb}}_{2}{\mathrm{Te}}_{3}\;\left(s\right)+2{\left({\mathrm{Me}}_{3}\mathrm{Si}\right)}_{3}\mathrm{Sb}\;\left(g\right)\;\to \;4\mathrm{Sb}\;\left(s\right)+3{\left({\mathrm{Me}}_{3}\mathrm{Si}\right)}_{2}\mathrm{Te}\;\left(g\right)$$

From the reaction, the theoretical weight change ratio by substituting one Sb_2_Te_3_ molecule (626.32 atomic mass units (amu)) with four Sb atoms (487.04 amu) must be 0.778, which is consistent with the experimental value of 0.782. This finding confirms that the proposed chemical substitution mechanism accounts for the formation of the *c*‐Sb film and that no additional Sb is deposited during the conversion process through precursor decomposition or physisorption. The Auger electron spectroscopy (AES) results of a 30‐nm‐thick *c*‐Sb film in Figure 2d further confirm this argument. Due to surface oxidation and contamination, O and C are detected on the film surface, which are diminished after sufficient Ar^+^ sputtering. Notably, the contents of Ge and Te are negligible, indicating that possible remnants from the sacrificial buffer layers do not exist, and that the current s‐ALD process yields a monatomic *c*‐Sb film with high chemical purity.

To additionally verify the purity of the *c*‐Sb film grown by the s‐ALD process, Raman spectroscopy was performed (Figure 2e). Before the conversion to *c*‐Sb, the characteristic E_g_ (in‐plane) vibration at 113.7 cm^−1^ and the A_g_ (out‐of‐plane) vibrations at 69.6 cm^−1^, 166.7 cm^−1^ of rhombohedral Sb_2_Te_3_ were observed in the spectra (green), indicating high crystallinity of the aligned *c*‐Sb_2_Te_3_ buffer layer.^[^ ^36^, ^37^ ^]^ After the s‐ALD, all peaks from the *c*‐Sb_2_Te_3_ disappeared, and the characteristic E_g_ (in‐plane) vibration at 119.1 cm^−1^ and the A_1g_ (out‐of‐plane) vibration at 153.8 cm^−1^ of rhombohedral Sb emerged.^[^ ^28^, ^38^ ^]^ The peaks of Sb_2_O_3_ were also not found, indicating the possible surface oxide layer has an amorphous structure.^[^ ^39^ ^]^ To further certify the chemical purity of the *c*‐Sb film, samples were analyzed by X‐ray photoelectron spectroscopy (XPS). Figure 2f shows the XPS result of the 7‐nm‐thick *c*‐Sb film deposited by the 3‐step s‐ALD on a SiO_2_ substrate after 1.5 min of Ar^+^ ion sputter‐cleaning, which indicates a monatomic singular bonding state of Sb (Sb‐Sb, Sb 3d_3/2_ = 537.6 eV, Sb 3d_5/2_ = 528.2 eV) without traces of Sb‐Te or Sb‐O bonds.^[^ ^40^, ^41^ ^]^ Figure S8 (Supporting Information) shows the XPS results of all the sacrificial layers and the resultant films used in the whole s‐ALD process. The constituent elements' XPS peaks and the appearance/disappearance of the peaks through chemical substitution of the elements manifest the chemical homogeneity of the respective films and the clarity of the conversion process of the s‐ALD.

## 4 Wafer‐Scale Uniformity and Conformality

**Figure 3:**
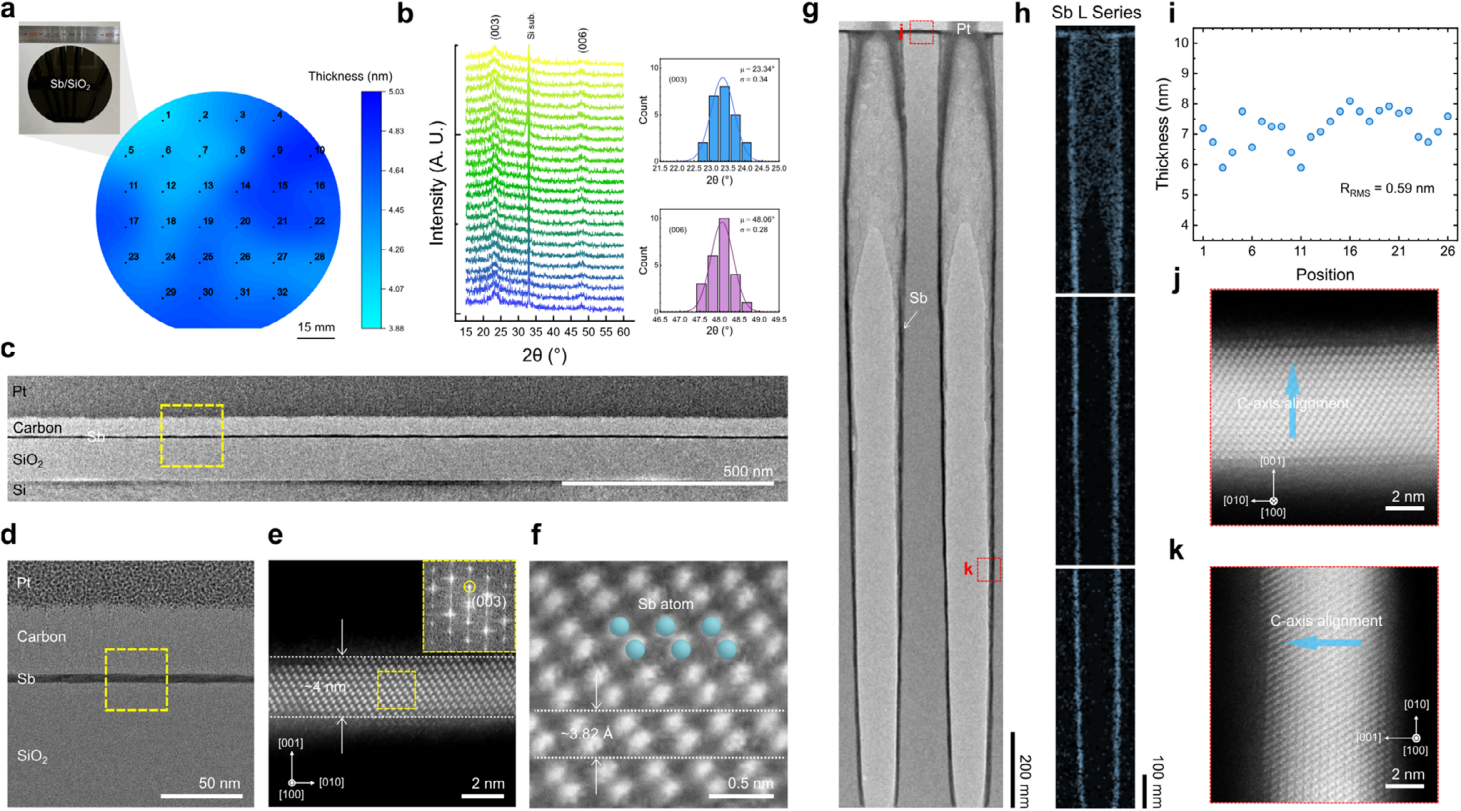
Wafer‐scale uniformity and conformality of s‐ALD *c*‐Sb films. a, b) Wafer‐scale uniformity of thickness (a) (optical image at inset) across a 4‐inch wafer and crystallinity (b), demonstrated by θ‐2θ XRD patterns with uniform distributions of Sb (003), (006) peaks. c, d) Cross‐sectional TEM images of s‐ALD *c*‐Sb deposited on a SiO_2_ substrate. e, f) Cross‐sectional HAADF‐STEM images of the s‐ALD *c*‐Sb displaying the characteristic bilayer structure of Sb. In figures c–f, the magnified view of the yellow boxed region from the preceding image is shown in the following alphabetical figure. g, h) Cross‐sectional TEM image (g) and TEM‐EDS mapping image (h) of s‐ALD *c*‐Sb deposited on a HAR (1:25) hole structure. i) Thickness of *c*‐Sb films measured on 26 positions of the hole structure. j, k) Cross‐sectional HAADF‐STEM images of the s‐ALD *c*‐Sb on the planar (j) and sidewall regions (k) of the hole structure. The regions where the STEM images were acquired are indicated by red boxes in Figure 3g.

**Figure** **3**a illustrates the thickness uniformity of the *c*‐Sb film across the 4‐inch SiO_2_ wafer. The optical microscope image of the deposited *c*‐Sb film displays uniform color over the whole wafer. The layer density of the film was measured across 32 points on the wafer using XRF, and then divided by the film's density, as measured via XRR, to calculate the film thickness. The results show an average thickness of ≈4.48 nm and a standard deviation of ≈0.30 nm with a low non‐uniformity of 6.70 %, calculated as 100·standard deviation/average thickness. Also, XRD analysis was conducted on 24 points of the wafer to confirm the uniform crystallinity of the *c*‐Sb film (Figure 3b). In all the θ‐2θ XRD scans, the Sb (003) plane (23.4°) and the Sb (006) plane (48.2°) were detected, suggesting that the (00*l*)‐alignment of the film was maintained throughout the whole wafer. Figure S9 (Supporting Information) shows the SEM images of the respective positions, which display smooth surface morphology at all points. Figure 3c–f shows the cross‐section transmission electron microscopy (TEM) images of the *c*‐Sb film deposited on a SiO_2_ substrate and atomic‐scale images with high‐angle annular dark‐field scanning transmission electron microscopy (HAADF‐STEM). The ultrathin *c*‐Sb film uniformly covers the SiO_2_ substrate (Figure 3c), as demonstrated again in the magnified STEM images. The bilayer structure of Sb (Sb‐Sb) is visible, with the shorter interlayer Sb bond and the longer intralayer Sb bond (Figure 3e). Figure 3f shows a magnified image, where the thickness of the bilayer is calculated to be 3.82 Å, consistent with previous studies.^[^ ^28^, ^42^ ^]^ The bilayer structure of Sb is repeated along the c‐axis of the crystal lattice, which is aligned vertically to the film substrate.

Figure 3g is the TEM image of the *c*‐Sb film deposited on the HAR (1:25) nanoscale hole structure, demonstrating conformal and uniform film growth. Figure 3h shows the TEM energy dispersive X‐ray spectroscopy (TEM‐EDS) images of the top, middle, and bottom parts of the hole. The TEM‐EDS results of the Sb L series verify conformal film deposition even on the sidewalls of the hole structure. Furthermore, Ge and Te signals are absent, demonstrating the chemical purity of the *c*‐Sb film (Figure S10, Supporting Information). Figure 3i shows the thicknesses of the deposited *c*‐Sb film measured from the TEM image at 26 points across the HAR structure. The magnified TEM images indicating the points where the thickness was measured are shown in Figure S11 (Supporting Information). The calculated R_RMS_ from the thicknesses of the measured points is 0.59 nm, indicating the smoothness of the *c*‐Sb film deposited by the three‐step s‐ALD, consistent with the measured value of 0.38 nm at the planar substrate. Figure 3j and k are the HAADF‐STEM images of the *c*‐Sb film deposited on the upper planar region and the lower sidewall region of the hole, respectively, indicated by the red box in Figure 3g. Interestingly, in both the planar region and the sidewall region, the c‐axis of *c*‐Sb is aligned vertically to the surface, indicating that the (00*l*)‐alignment of the *c*‐Sb film is maintained even over the sidewall region of the hole structure. These results demonstrate that the s‐ALD *c*‐Sb film possesses exceptional wafer‐scale uniformity and conformality. Combined with the relatively low deposition temperature (≤ 220 °C) described in the previous section, the s‐ALD process is fully compatible with standard complementary metal‐oxide‐semiconductor (CMOS) technologies and provides a reliable pathway for implementing ultrathin Sb films for next‐generation monolithic 3D integration architectures.^[^ ^43^ ^]^

## 5 Mechanism of the Conversion from Sb_2_Te_3_ to Sb

**Figure 4:**
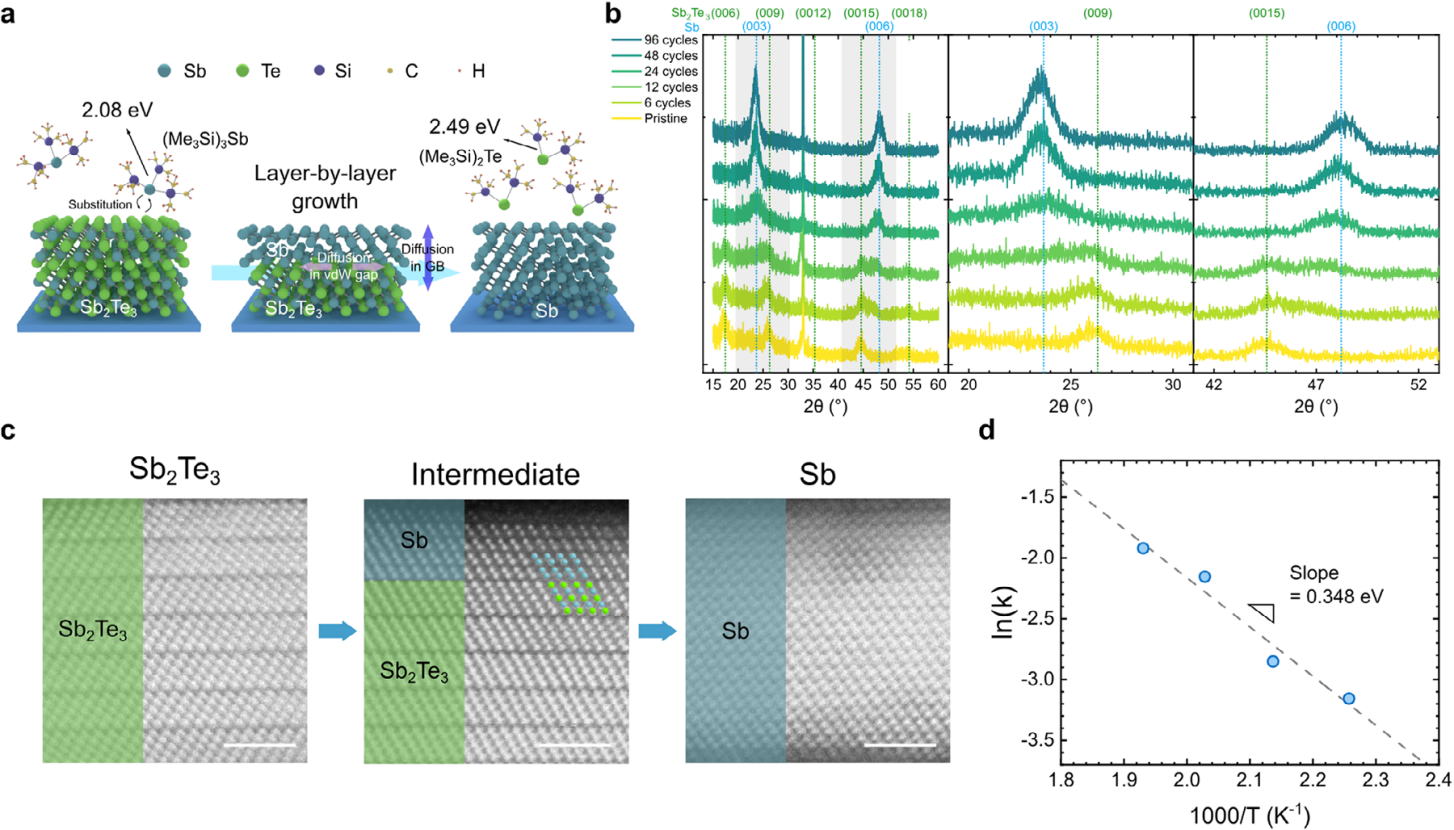
Mechanism analysis of s‐ALD from *c*‐Sb_2_Te_3_ to *c*‐Sb. a) Schematic illustration of layer‐by‐layer transformation from *c*‐Sb_2_Te_3_ to *c*‐Sb. The bonding preference between Me_3_Si‐ and Te drives the reaction forward, and enhanced atomic diffusion through the aligned structure facilitates efficient conversion. b) Evolution of θ‐2θ X‐ray diffractogram of the *c*‐Sb_2_Te_3_/*c*‐Sb films during the s‐ALD process. c) Cross‐sectional STEM images of the pristine *c*‐Sb_2_Te_3_ (left), intermediate *c*‐Sb_2_Te_3_/*c*‐Sb (center) and final *c*‐Sb (right) film. Scale bar: 2 nm. d) Arrhenius plot of the reaction rate constant.

This section provides an in‐depth analysis of the mechanism underlying the conversion from *c*‐Sb_2_Te_3_ to *c*‐Sb. **Figure** **4**a schematically shows the overall process of the s‐ALD from *c*‐Sb_2_Te_3_ to *c*‐Sb. As previously mentioned, the Te atoms in the *c*‐Sb_2_Te_3_ film are chemically substituted by the Sb atoms originating from the adsorbing (Me_3_Si)_3_Sb precursor, and thus the (00*l*)‐aligned *c*‐Sb_2_Te_3_ is transformed into a (00*l*)‐aligned *c*‐Sb. The driving force behind the substitution is the stronger bonding strength between Te atoms and the Me_3_Si‐ ligand than Sb, which can be explained qualitatively by the hard and soft Lewis acid and bases (HSAB) theory. HSAB theory states that hard (soft) acids prefer to bond with hard (soft) bases.^[^ ^44^ ^]^ Me_3_Si‐ is a hard Lewis acid, and since Te is a relatively harder Lewis base than Sb, it preferentially binds to the Me_3_Si‐ ligand, resulting in the substitution of Te in the Sb_2_Te_3_ film with Sb.^[^ ^34^ ^]^ For a qualitative explanation, ab initio density functional theory (DFT) computations are performed to assess the spontaneity of the chemical substitution process. First, the bond dissociation energy (BDE) of Me_3_Si‐ with Te and Sb was calculated to be BDE_Me3Si‐Te_ = 2.49 eV and BDE_Me3Si‐Sb_ = 2.08 eV. This demonstrates that Te has a stronger bonding affinity to Me_3_Si‐ compared to Sb, consistent with the explanation by the HSAB theory. Moreover, the enthalpy and Gibbs free energy change of the substitution reaction have negative values as below.

(2) $${\mathrm{Sb}}_{2}{\mathrm{Te}}_{3}+2{\left({\mathrm{Me}}_{3}\mathrm{Si}\right)}_{3}\mathrm{Sb}\;\to \;4\mathrm{Sb}+3{\left({\mathrm{Me}}_{3}\mathrm{Si}\right)}_{2}\mathrm{Te}$$

(ΔH_493 K, 3.5 Torr_ = −218.99 kJ mol^−1^, ΔG_493 K, 3.5 Torr_ = −333.14 kJ mol^−1^)

This result verifies that the substitution reaction is thermodynamically favorable. Section S1 (Supporting Information) describes the detailed calculation results of the entire three‐step s‐ALD, including the first *a*‐GeTe to *c*‐Sb_2_Te_3_ conversion.

The intermediate structural transformation process was further examined by varying the ALD cycles of (Me_3_Si)_3_Sb precursor on the 7‐nm‐thick *c*‐Sb_2_Te_3_ film. As 96 cycles of (Me_3_Si)_3_Sb at 220 °C led to complete transformation to *c*‐Sb, intermediate cycle numbers of (Me_3_Si)_3_Sb pulses were applied (6, 12, 24, 48) and the resulting films were analyzed by XRD. Figure 4b shows the θ‐2θ XRD pattern of the respective films. Before the (Me_3_Si)_3_Sb pulse, only Sb_2_Te_3_ (00*l*) diffraction peaks are observed (bottom data, yellow), and only the Sb (00*l*) diffraction peaks are observed after 96 cycles of (Me_3_Si)_3_Sb pulse. Interestingly, the gradual transformation of *c*‐Sb_2_Te_3_ to *c*‐Sb is observed in the films when intermediate numbers of (Me_3_Si)_3_Sb pulses are applied. The second and third panels of Figure 4b present magnified views of the shaded regions in the first panel, showing the emergence of the Sb (003) and (006) peaks at 23.6° and 48.1°, respectively, and the simultaneous disappearance of Sb_2_Te_3_ (009) and (0015) peaks at 26.1° and 44.8°, respectively, with increasing cycle numbers. Figure S12 (Supporting Information) shows the GIXRD pattern of the respective *c*‐Sb_2_Te_3_/*c*‐Sb films, where no diffraction peaks of Sb_2_Te_3_ or Sb appear in the films. The absence of plane reflections in the GIXRD pattern and the exclusive appearance of Sb_2_Te_3_/Sb (00*l*) reflections in the θ‐2θ diffraction pattern indicate that the initial *c*‐Sb_2_Te_3_ film, intermediate films, and the fully converted *c*‐Sb film are all aligned with their (00*l*) planes lying parallel to the substrate.

The initial *c*‐Sb_2_Te_3_, intermediate *c*‐Sb_2_Te_3_/*c*‐Sb (12 cycles of (Me_3_Si)_3_Sb), and final *c*‐Sb film were analyzed by STEM to gain an atomistic insight into the transformation process during s‐ALD (Figure 4c). The HAADF‐STEM images of the initial *c*‐Sb_2_Te_3_ (left panel) and final *c*‐Sb films (right panel) exhibited atomic structures with the characteristic Te‐Sb‐Te‐Sb‐Te quintuple layer and Sb‐Sb bilayer, respectively. These findings corroborate the XRD results, indicating that the c‐axis of the films is aligned to the surface normal direction. Meanwhile, the intermediate film (center panel) exhibits two distinct regions. The upper region of the film displays the structure of elemental Sb, while the lower region shows the structure of Sb_2_Te_3_, which can be discerned by the characteristic layered features present in each region of the film. This indicates that the substitution process begins at the film surface and propagates towards the bottom interface in a layer‐by‐layer manner. Also, in‐plane orientations between the upper *c*‐Sb and lower *c*‐Sb_2_Te_3_ layers are consistent, indicating the local epitaxy between *c*‐Sb and *c*‐Sb_2_Te_3_ due to the small in‐plane lattice mismatch with a similar crystal structure. Therefore, these STEM images of *c*‐Sb_2_Te_3_/*c*‐Sb demonstrate that the top‐to‐bottom, layer‐by‐layer local epitaxial growth of *c*‐Sb occurs using aligned *c*‐Sb_2_Te_3_ as the sacrificial buffer, which leads to the conformal, uniform, (00*l*)‐aligned monatomic *c*‐Sb thin film (Figure S13, Supporting Information, shows a cross‐sectional STEM image of the *c*‐Sb film viewed along a different zone axis, clearly demonstrating the well‐preserved layered structure with distinct bilayer features after the complete conversion from *c*‐Sb_2_Te_3_).

The kinetics of the s‐ALD conversion process are analyzed by calculating the activation energy of the reaction. Figure 4d shows the Arrhenius plot of the reaction rate obtained from temperature‐dependent deposition (Figure 2a). Rate constants at each temperature are extracted from exponential fits to the experimental data, with detailed procedures provided in Section S2 (Supporting Information). The activation energy (E_a_), derived from the slope of the linear fit, is calculated to be 0.348 eV. Previous studies reported that the activation energy for the substitution reaction between Sb_2_Te_3_ and Sb is determined by Te diffusion through the grain boundaries of Sb_2_Te_3_ above 140 °C, with a value of 0.76 eV.^[^ ^34^ ^]^ Notably, the activation energy obtained in this study is significantly lower than the previously reported value. This discrepancy may indicate that the aligned film crystalline structure facilitates the atomic diffusion. In 2D layered materials, atomic diffusion along van der Waals gaps is energetically more favorable than diffusion through the covalently bonded lattice.^[^ ^45^, ^46^ ^]^ Also, the parallel van der Waals gaps of the current film can connect the grain boundaries and further provide a facile pathway for diffusion of atoms, leading to a lower energy barrier. In this case, the atoms preferentially diffuse horizontally through the parallelly aligned van der Waals gaps to the grain boundaries, and subsequently migrate vertically through the grain boundaries, which have lower energy barriers than those across the 2D layers. This combined diffusion network enables atoms to reach the film surface efficiently, which leads to complete substitution of Te into Sb throughout the entire film.

## 6 Sb monatomic Phase Change Memory

**Figure 5:**
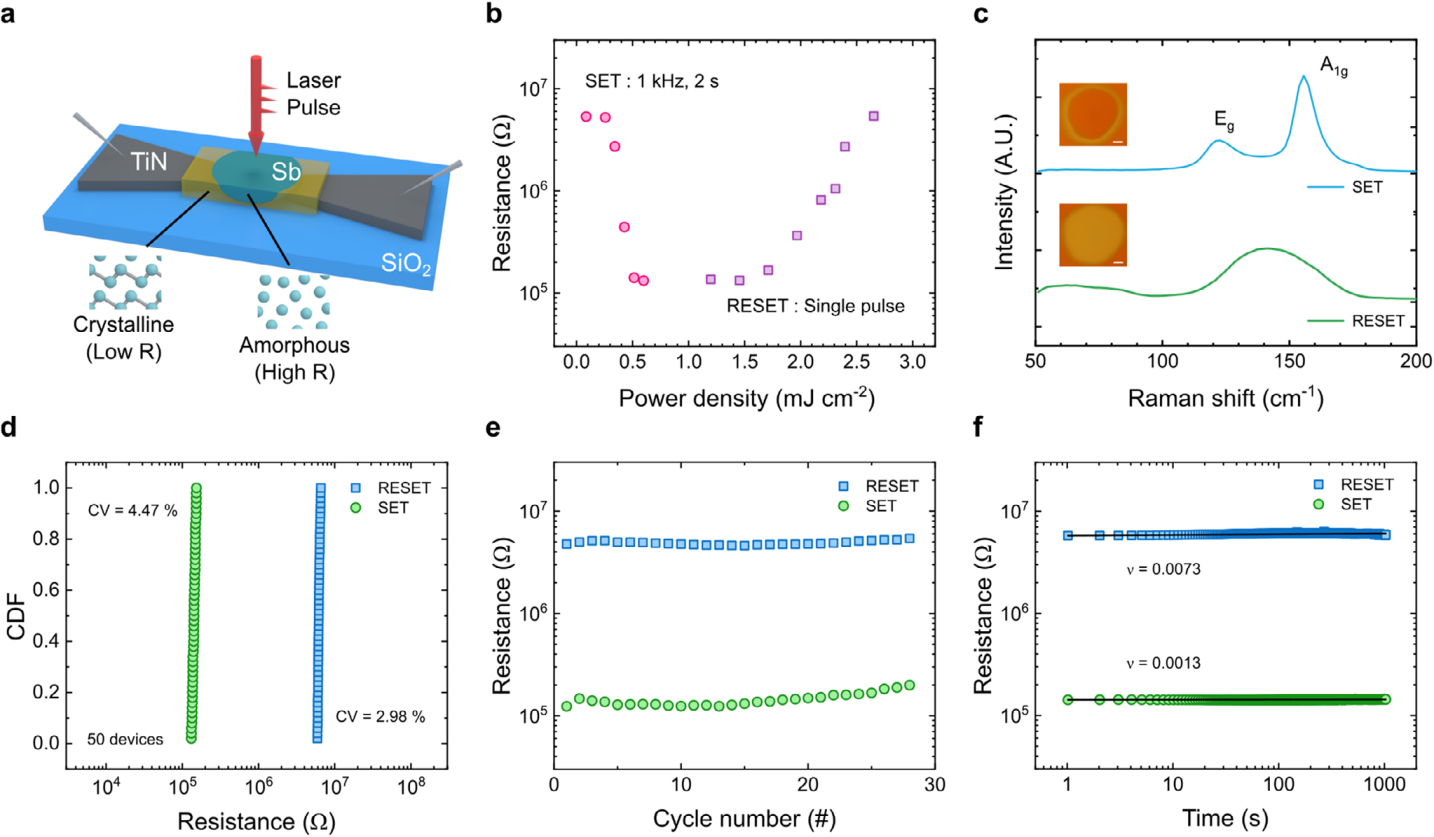
Ultrafast phase change memory characteristics of s‐ALD Sb films. a) PCM device configuration demonstrating reversible resistance switching via femtosecond laser pulses (≈220 fs). b) Resistance‐fluence characteristics of the Sb PCM device. c) Raman spectra of the crystalline SET and amorphous RESET states. (inset) Optical microscope images of the film surface where the Raman spectra were acquired. (scale bar: 100 µm) d) Cumulative distribution function of resistance values of 50 devices of the SET and RESET states. e) Cycling performance of the Sb PCM device. f) Resistance drift characteristics of the SET and RESET states.

The exceptional uniformity and precise thickness controllability achieved through the s‐ALD process enable the realization of high‐quality Sb PCM devices. These devices, which cannot be obtained with conventional ALD techniques, may exhibit faster switching speeds compared to conventional multi‐component phase change materials, such as Ge_2_Sb_2_Te_5_.^[^ ^47^ ^]^ The standard method to evaluate PCM switching speed is to utilize electrical pulses. However, it is challenging to decrease the pulse width to under several nanoseconds due to inherent circuit parasitics, such as resistance‐capacitance (RC) delay.^[^ ^48^ ^]^ To overcome such speed limitations of electrical tests and to evaluate the fast switching speed of Sb, optical switching using ultrafast laser pulses was employed. It has been reported that this method enables operation time down to the femtosecond range and opens possibilities for optoelectronic hybrid memory applications.^[^ ^49^, ^50^, ^51^ ^]^ **Figure** **5**a shows the schematic diagram of the lateral‐type device incorporating the 5 nm s‐ALD *c*‐Sb film that is optically switched and electrically read. Two 50‐nm‐thick TiN electrodes with a gap (width and length of 50 and 30 µm) are patterned on the SiO_2_/Si substrate. Then, the 5 nm *c*‐Sb film was grown by the three‐step s‐ALD, and a 10 nm Al_2_O_3_ film was deposited on the test structure as the passivation layer by sputtering. Figure S14 (Supporting Information) shows the detailed fabrication process. The lab‐built testing system was used to measure the memory performance of the devices, described in detail in the Experimental Section and Figure S15 (Supporting Information). The resistance was measured by probing two TiN electrodes after the femtosecond laser pulse‐induced phase change operation (≈220 fs length at 1030 nm wavelength). Although the nominal laser spot size (1.725 mm in diameter) is much larger than the electrode gap region, the Gaussian intensity profile of the laser beam creates a localized active switching region concentrated near the center of the electrode gap, as illustrated in Figure 5a.

Figure 5b presents the resistance‐laser fluence characteristics for SET (switching from the off state to the on state) and RESET (vice versa) operations. To initially transform the as‐deposited *c*‐Sb film into a high‐resistance state, a single laser pulse with ≈220 fs duration and 2.65 mJ cm^−2^ fluence was applied. This operation induced the RESET of the device, and the resistance increased to ≈5 × 10^6^ Ω. To induce the SET operation of the device, the laser pulse duration was extended to 10 ps, which is the longest limit of the laser setup. However, the SET state could not be achieved with a single laser pulse. This appears to be due to the intrinsic SET switching speed of Sb, which typically requires several hundred picoseconds for complete crystallization.^[^ ^47^ ^]^ Therefore, the ≈220 fs laser pulse was repeatedly shone at 1 kHz for 2 s, with the increasing laser fluence, indicated by the pink circle data in Figure 5b. The device started to show an abrupt decrease in the resistance from ≈0.3 mJ cm^−2^ and recovered the initial low value (≈1 × 10^5^ Ω) at ≈0.51 mJ cm^−2^. Due to these experimental limitations, the SET speed of this Sb film‐based PCM cannot be accurately estimated. However, the RESET can be reliably achieved with a single pulse when the laser fluence is increased to over ≈2 mJ cm^−2^, and it recovered the off resistance (≈5 × 10^6^ Ω) at ≈2.65 mJ cm^−2^ (purple square). Therefore, a much shorter RESET time (≈220 fs) compared to conventional PCM materials such as Ge_2_Sb_2_Te_5_ (≈13 ps)^[^ ^49^ ^]^ can be achieved from the elemental Sb PCM.

Figure 5c shows the Raman spectra of Sb films measured in SET and RESET states, which confirms that the resistance change characteristics of the device are due to amorphization and crystallization. In the SET state, characteristic peaks are observed at 155 cm^−1^ and 120 cm^−1^, corresponding to the A_1g_ and E_g_ vibrational modes of the rhombohedral Sb film. In contrast, the RESET state shows the disappearance of these crystalline peaks. It shows a broad peak in the 120‐160 cm^−1^ region characteristic of an amorphous Sb,^[^ ^52^ ^]^ confirming phase transformation between amorphous and crystalline states without the formation of other phases. The Raman spectra of the films in devices with intermediate resistances between those of the SET and RESET states also indicated the coexistence of crystalline and amorphous phases (Figure S16, Supporting Information). The inset figures of Figure 5c show the optical microscope images of the Sb films used for Raman analysis of SET and RESET states, which display distinct color changes between the two states. This indicates that the *c*‐Sb films deposited by s‐ALD also have potential as tunable nonvolatile optical materials.^[^ ^7^, ^8^ ^]^

Figure 5d presents the cumulative distribution function of resistance values for SET and RESET states measured across 50 devices. The statistical analysis reveals exceptional device uniformity with coefficient of variation (100 standard deviation/mean) values of 4.47 % for the SET state and 2.98 % for the RESET state. These low variation coefficients demonstrate the high reliability of devices and the excellent uniformity of *c*‐Sb films deposited by the s‐ALD, making it highly suitable for large‐scale fabrication. Figure 5e shows the cycling performance of the Sb PCM device over repeated SET/RESET cycling operations. An on/off ratio of ≈50 was maintained for over 30 cycles, indicating that repeated reversible phase change by femtosecond laser pulses is possible. When the cycling was performed over 50 cycles, the device stuck at the RESET state due to the agglomeration of the Sb film. The limited cycling performance can be attributed to the ultrathin nature of the active layer, which may be more susceptible to interface degradation and void formation during repeated switching.^[^ ^53^ ^]^ Still, to the best of the authors’ knowledge, this is one of the few demonstrations of the cycling performance in PCM using elemental Sb. Future optimization of the laser operating scheme^[^ ^54^, ^55^ ^]^ and device structure^[^ ^56^ ^]^ together with interface engineering^[^ ^57^ ^]^ and alloying strategies^[^ ^51^ ^]^ would significantly improve the device endurance characteristics while maintaining the advantages of s‐ALD processing, which is beyond the scope of the present work.

Figure 5f illustrates the drift characteristics of the SET and RESET states. In PCM devices, the drift coefficient generally follows a power law relationship given by: R(t) = R_0_ × (t/t_0_)^ν^, where R(t) is the resistance at time t, R_0_ is the initial resistance, t_0_ is the reference time, and ν is the drift coefficient.^[^ ^58^ ^]^ The SET state represents the crystalline phase with a stable atomic arrangement, while the RESET state corresponds to the metastable amorphous phase that tends to relax over time structurally. Particularly, for the Sb thin film, due to its strong crystallization tendency, spontaneous recrystallization of the amorphous phase occurs easily at film thicknesses above 6 nm.^[^ ^6^ ^]^ However, the drift coefficient (ν) for SET and RESET states for the current devices showed extremely low values of 0.0013 and 0.0073, respectively, which are lower than the previously reported values for the multi‐component PCM by one to two orders of magnitude.^[^ ^59^, ^60^, ^61^ ^]^ This indicates that structural relaxation or spontaneous crystallization is minimal in the amorphous phase. Thus, these ultralow drift coefficients demonstrate the advantage of using s‐ALD *c*‐Sb films, which eliminates compositional instabilities and could further enable reliable multibit programming applications.^[^ ^5^ ^]^

To explicitly demonstrate the potential of the conformality of s‐ALD Sb, vertical‐type PCM devices were fabricated by etching the SiO_2_ substrate before depositing and patterning the electrode and active layer (Figure S17, Supporting Information). The vertical‐type PCM devices exhibited performance comparable to planar configurations, achieving ultrafast switching with femtosecond laser pulses, stable cycling over 30 cycles, and ultralow drift coefficients (0.0033 for RESET, 0.0020 for SET), confirming that the s‐ALD approach enables practical implementation of vertical PCM architectures, which are crucial for next‐generation high‐density memory devices.

## 7 Conclusion

This study presents a novel sacrificial atomic layer deposition approach that feasibly overcomes the fundamental limitations of conventional ALD for producing ultrathin *c*‐Sb films. The *c*‐Sb film could be grown in a layer‐by‐layer manner through chemical substitution of the Te atoms in the c‐axis‐oriented *c*‐Sb_2_Te_3_ buffer layer, which was achieved by replacing the Ge atoms in another sacrificial *a*‐GeTe layer with Sb atoms, with the Sb atoms from the Sb‐precursor. This specific process leads to a monatomic *c*‐Sb film with excellent uniformity and a low surface roughness of 0.38 nm at a minimum thickness of ≈4 nm. Analysis of the *c*‐Sb film through XRF, Raman spectroscopy, XPS, AES and TEM‐EDS reveals the high chemical purity of the deposited film. The crystallographic resemblance between Sb_2_Te_3_ and Sb enables the local epitaxial growth of layered Sb with its c‐axis oriented perpendicularly to the substrate surface. Wafer‐scale uniform growth of ultrathin (≈4 nm) *c*‐Sb films and superior conformality on complex high aspect ratio structures (1:25) at low temperature (≤ 220 °C) were demonstrated, establishing CMOS‐compatibility and suitability for monolithic 3D integration architectures. The substitution mechanism is driven by the stronger bonding affinity of Te to Me_3_Si‐ ligands compared to Sb, with the aligned crystal structure providing enhanced diffusion pathways that decrease the activation energy barrier. Phase change memory devices fabricated with 5‐nm‐thick s‐ALD‐deposited *c*‐Sb films exhibited ultrafast switching capabilities with femtosecond laser pulses, opening new possibilities for high‐speed memory applications. Furthermore, the exceptional device‐to‐device uniformity directly reflects the superior thickness controllability and morphological consistency of the s‐ALD process, which is advantageous for large‐scale manufacturing. Therefore, this novel ALD methodology provides a promising approach for depositing monatomic materials with excellent conformality and wafer‐scale uniformity. The achieved breakthrough in ultrathin film deposition paves the way for the realization of next‐generation electronic and photonic devices.

## 8 Experimental Section

### Film Deposition

The films were deposited in an 8‐inch wafer scale showerhead‐type atomic layer deposition (ALD) reactor (CN‐1, Plus‐200). Ge^II^NMe_2_[(N*i*Pr)_2_CNMe_2_], (Me_3_Si)_2_Te, Sb(OEt)_3_ and (Me_3_Si)_3_Sb were used as the Ge, Te, and Sb precursors, respectively. Me, *i*Pr and Et refer to the methyl (CH_3_), isopropyl ((CH_3_)_2_CH‐), and ethyl (C_2_H_5_) groups, respectively. The precursors were kept in a bubbler‐type canister, where they were heated to 65 °C, 30 °C, 40 °C, and 40 °C, respectively, to provide appropriate vapor pressures suitable for ALD. NH_3_ gas was co‐injected with (Me_3_Si)_2_Te to enhance the precursor reactions. The *a*‐GeTe, *c*‐Sb_2_Te_3_, and *c*‐Sb were deposited at 130, 170, 170–245 °C, respectively, at working pressures of 3.3–3.7 Torr. Dry oxidized 100‐nm‐thick SiO_2_/Si was used as a substrate, which was ultrasonicated for 10 minutes in acetone and isopropyl alcohol to remove organic contaminants before deposition. More specific details of the film deposition process were provided in Section S1 (Supporting Information).

### Film Characterization

The layer density of films was measured by X‐ray fluorescence spectroscopy (Thermo Scientific, QUANT'X EDXRF) in a standard‐based method for accurate calibration. The film bulk density was measured by X‐ray reflectivity (PANalytical, X'Pert PRO MPD) using a Cu Kα X‐ray tube. The XRR results were fitted with X'Pert Reflectivity software by PANalytical. The thickness of the films was calculated by dividing the layer density by the bulk density. The surface morphology of the films was observed by scanning electron microscopy (SEM, Hitachi, S‐4800) and atomic force microscopy (AFM, Park Systems, NX10). AFM measurements were performed using a silicon cantilever with a high resonant frequency (≈330 kHz) and a nominal spring constant of ≈42 N m^−1^. The probe featured a backside aluminum reflective coating, with a typical tip length of 10–15 µm and a nominal tip radius of < 10 nm. Plane fitting was applied to remove background tilt. The film resistivity was measured by a 4‐point probe (AIT, CMT‐SR2000N). Hall measurement (BIO‐RAD, HL5500PC) was used to measure the film resistivity and electrical mobility. Hall measurements were conducted using the van der Pauw method, where Ni electrical contacts were formed by e‐beam evaporation (Sorona, SRN‐200). Raman spectroscopy (Horiba, LabRAM HR Evolution) was adopted to observe atomic vibrations. A 532 nm excitation laser with 100 mW power attenuated by a 3.2 % neutral density filter was used, and a 100× objective lens was employed to focus the beam on the sample surface. X‐ray diffraction (PANalytical, X'Pert PRO MPD) with a Cu Kα X‐ray tube was used to examine film crystallinity and texture. X‐ray photoelectron spectroscopy (ULVAC‐PHI, VersaProbe 3) was used to analyze the chemical bonding states using a monochromated Al Kα source (1486.6 eV, 27.3 W) with a 100 µm beam diameter. Spectra were acquired with a pass energy of 112 eV, and the binding energies were referenced to the adventitious carbon C 1s peak at 284.8 eV. Auger electron spectroscopy (ULVAC‐PHI, PHI‐700) was used to detect impurity elements in the film. Spectra were acquired with an electron beam energy of 5 kV and a target current of 10 nA using a cylindrical mirror analyzer. Spherical aberration‐corrected transmission electron microscopy/scanning transmission electron microscopy (JEOL, JEM‐ARM200F) was used to observe conformal film deposition on a complex nanoscale structure and to analyze the aligned crystalline structure of Sb on an atomic scale. Energy dispersive spectroscopy was adopted to verify the chemical purity of the deposited film. The TEM specimens were prepared with a focused ion beam (Thermo Fisher Scientific, Helios G4).

### Device Fabrication and Characterization

Lateral‐type phase change memory devices were fabricated by photolithography (Figure S10, Supporting Information). 50 nm TiN was sputtered (Sorona, SRN‐120) on a SiO_2_ substrate and patterned by photolithography and dry etching as a lateral‐type bottom electrode. Then, a 5 nm *c*‐Sb film was deposited by s‐ALD as an active layer, and a 10 nm Al_2_O_3_ film was deposited ex situ by sputtering (Sorona, SRN‐120) as a capping layer onto the lateral‐type bottom TiN electrode. Then the active region of the device was defined by photolithography and dry etching. The device testing system is lab‐built as shown in Figure S15 (Supporting Information), where the SET/RESET operations were performed by a femtosecond laser (Light Conversion, PHAROS PH2‐20W) with a laser wavelength of 1030 nm, spot size of 1.725 mm and a single pulse width of 220 fs. The changes in resistance of the film/device were measured by an electrical detection module using a semiconductor parameter analyzer (Keithley, DAQ6510).

### First‐Principle Calculations

Ab initio density functional theory computations by the Gaussian 09 software were utilized to explain the spontaneity of the s‐ALD reactions. The BDE of the precursor molecules and the Gibbs free energy change of reactions were calculated. The energies and geometries of the relevant molecules were calculated using Becke's three‐parameter hybrid functional combined with the Lee, Yang, and Parr (LYP) correlation functional (B3LYP). The double‐zeta polarized 6‐31G(d) basis set was used for Ge, Si, C, O, and H. The balanced polarized triple‐zeta def2‐TZVPD basis set was used for Te and Sb.

## Conflict of Interest

The authors declare no conflict of interest.

## Supporting information



Supporting Information

## Data Availability

The data that support the findings of this study are available from the corresponding author upon reasonable request.
