# Supplementary material for: Ultrathin Monatomic Antimony Films by Sacrificial Atomic Layer Deposition for Phase Change Memory
Source: Adv Mater. 2025 Nov 29;38(8):e19924. doi: 10.1002/adma.202519924 (PMC12878807; doi:10.1002/adma.202519924)
Supplement: Supplementary file 1 — Supporting Information [file ADMA-38-e19924-s001.docx]

**Supporting Information for**

**Ultrathin Monatomic Antimony Films by Sacrificial Atomic Layer Deposition for Phase Change Memory**

*Gwangsik Jeon^1,†^, Sangmin Jeon^1,†^, Seunghwan Lee^2^, Jeong Woo Jeon^1^, Wonho Choi^1^, Byongwoo Park^1^, Sungjin Kim^1^, Chanyoung Yoo^3^, Hyejin Jang^2^ and Cheol Seong Hwang^1,^**

^1^Department of Materials Science and Engineering, and Inter-University Semiconductor Research Center, Seoul National University, Seoul, 08826, Republic of Korea

^2^Department of Materials Science and Engineering, Seoul National University, Seoul, 08826, Republic of Korea

^3^Department of Materials Science and Engineering, Hongik University, Seoul, 04066, Republic of Korea

^†^ These authors contributed equally to this work.

* Corresponding author (e-mail: cheolsh@snu.ac.kr)

**Table of contents**

- **Section 1. The three-step s-ALD process of the monatomic Sb film**
- **Section 2. Analysis of the reaction kinetics in s-ALD from Sb_2_Te_3_ to Sb**
- **Table S1**
- **Figures S1-S17**
- **References**

**Section 1. The three-step s-ALD process of the monatomic Sb film**

Figure S1 displays the overall process of the three-step s-ALD for Sb film deposition. The sacrificial amorphous GeTe (*a*-GeTe) buffer film is deposited by the sequential injection of Ge^II^NMe_2_[(N*i*Pr)_2_CNMe_2_] and (Me_3_Si)_2_Te precursors at 130 ℃ (Me = CH_3_, *i*Pr = (CH_3_)_2_CH-). NH_3_ is co-injected with the Te precursor to enhance the reaction and film deposition. The optimal pulse/purge time for the *a*-GeTe deposition was determined to be 3 s – 15 s – 2 s – 15 s (Ge pulse – Ar purge – Te/NH_3_ pulse – Ar purge).^[1]^ The amorphous nature of the as-deposited GeTe film is advantageous for obtaining continuous thin films even at low thickness. Subsequently, the deposition of uniform aligned crystalline Sb_2_Te_3_ (*c*-Sb_2_Te_3_) occurs by the sequential injection of Sb(OEt)_3_ and (Me_3_Si)_2_Te precursors on *a*-GeTe buffer film at 170 ℃ (Et = C_2_H_5_). NH_3_ is again co-injected with the Te precursor to enhance deposition. The optimal pulse/purge time for the *c*-Sb_2_Te_3_ deposition was 2 s – 15 s – 2 s – 15 s (Sb pulse – Ar purge – Te/NH_3_ pulse – Ar purge). During this process, the Ge atoms in the underlying film are chemically substituted with Sb atoms.^[2]^ Finally, the complete conversion to monatomic crystalline Sb (*c*-Sb) is achieved by injecting the (Me_3_Si)_3_Sb precursor and purging out by-products. During this process, Te atoms in the film are chemically substituted with Sb atoms. Film conversion was tested at the ALD chamber temperature of 170 ~245 ℃, and 220 ℃ was determined to be the optimal temperature. The optimal pulse/purge time for the Sb deposition was determined to be 5 s – 20 s (Sb pulse – Ar purge).

The Chemical/thermodynamic spontaneity of each chemical substitution process in s-ALD (GeTe → Sb_2_Te_3_, Sb_2_Te_3_ → Sb) was analyzed. HSAB theory is utilized to describe the driving force behind such reactions since it is particularly useful for the case of transition metal chemistry. In the first step of the sacrificial ALD, Ge in the *a*-GeTe film is substituted with Sb in the Sb(OEt)_3_ precursor. -OEt is a hard base, thus prefers to bond with a hard acid. The Lewis acidity of Ge is harder than that of Sb; thus, -OEt prefers to bond with Ge than with Sb. This tendency acts as a driving force for the chemical substitution, and consequently, *c*-Sb_2_Te_3_ is deposited with no remaining Ge left on the film. Likewise, in the second step of the s-ALD, Te in the *c*-Sb_2_Te_3_ film is substituted with Sb in the (Me_3_Si)_3_Sb precursor. In this case, Me_3_Si- is a strong Lewis acid; thus, it prefers to bond with a strong base. The Lewis basicity of Te is stronger than that of Sb, so that Me_3_Si-Te is preferred over Me_3_Si-Sb. Thus, substitution is spontaneous, and the film is completely transformed to *c*-Sb without any residual Te remaining in the film.

As a more quantitative approach, the authors adopted *ab initio* density functional theory (DFT) computations to scrutinize the spontaneity of each step of the chemical substitution (Table S1). Firstly, the bond dissociation energy (BDE) of the precursor molecules was calculated to confirm the bonding preference between elements and ligands. For the case of -OEt, the BDE of Ge-OEt (3.78 eV) was higher than that of Sb-OEt (2.78 eV), thus agreeing with the HSAB theory that -OEt tends to bond with Ge rather than Sb. For the case of Me_3_Si-, the BDE of Me_3_Si-Te (2.49 eV) was higher than that of Me_3_Si-Sb (2.08 eV), again corroborating the previous chemical explanation.

Moreover, the Gibbs free energy change was calculated for each of the cases of the substitution reaction to exactly verify the spontaneity of the reactions. The chemical equation for the first substitution reaction can be written as follows.

3Ge + 4Sb(OEt)_3_ → 4Sb + 3Ge(OEt)_4_ (S1)

The enthalpy and Gibbs free energy change of the reaction were calculated to be

Δ H _443 K, 3.5 Torr_ = -733.53 kJ mol^-1^, Δ G _443 K, 3.5 Torr_ = -696.60 kJ mol^-1^

, where the negative value of Δ G indicates the spontaneity of the reaction.

Likewise, the chemical equation for the second substitution reaction can be written as,

Sb_2_Te_3_ + 2(Me_3_Si)_3_Sb → 4Sb + 3(Me_3_Si)_2_Te (S2)

The enthalpy and Gibbs free energy change of the reaction were calculated to be

Δ H _493 K, 3.5 Torr_ = -218.99 kJ mol^-1^ ,Δ G _493 K, 3.5 Torr_ = -333.14 kJ mol^-1^.

Again, the negative value of Δ G verifies that the reaction is spontaneous.

**Section 2. Analysis of the reaction kinetics in s-ALD from Sb_2_Te_3_ to Sb**

The activation energy of the s-ALD conversion process was determined by analyzing the temperature-dependent results shown in Figure 2a. The experimental data at each temperature were fitted using an exponential decay function:

C(t) = C_0_ + (C_init_ - C_0_) × exp(-kt) (S3)

, where C(t) is the Sb concentration at time t (equivalent to the number of pulses), C_0_ is the final equilibrium concentration, C_init_ is the initial concentration, k is the reaction rate constant, and t represents the number of (Me_3_Si)_3_Sb pulses. The rate constant k extracted from the exponential fitting has different physical interpretations depending on the dominant mechanism.

For first-order chemical reactions, k represents the reaction rate constant, which follows the Arrhenius equation:^[3]^

k = A × exp(-E_a_/RT) (S4)

Also, for diffusion-controlled processes, k represents the effective diffusion coefficient, which also exhibits Arrhenius behavior:^[4]^

D_eff_ = D_0_ × exp(-E_a_/RT) (S5)

In both cases, the temperature dependence follows the same exponential relationship, allowing the extraction of activation energy through Arrhenius analysis. Since the s-ALD conversion process involves both surface chemical reactions and bulk diffusion of Te atoms through the film, both mechanisms could contribute to the overall kinetics. The common exponential form allows determination of an effective activation energy that represents the rate-limiting step, regardless of whether it is reaction-limited or diffusion-limited.

The rate constants extracted at different temperatures (170, 195, 220, and 245 °C) were plotted in Arrhenius coordinates (ln(k) vs. 1/T) to determine the activation energy from the slope according to:

ln(k) = ln(A) - E_a_/(RT) (6)

, where A is the pre-exponential factor, Eₐ is the activation energy, R is the gas constant, and T is the absolute temperature (Figure 4d).

**Table S1**

**Table S1.** The summarized results of ab initio density functional theory computations of the second and third steps of the chemical substitution in the three-step s-ALD.

|  | **Step 2 : GeTe → Sb_2_Te_3_** | **Step 3 : Sb_2_Te_3_ → Sb** |
| --- | --- | --- |
| BDE | Ge-OEt (3.78 eV) > Sb-OEt (2.78 eV) | Me_3_Si-Te (2.49 eV) > Me_3_Si-Sb (2.08 eV) |
| Reaction | 3Ge + 4Sb(OEt)_3_  → 4Sb + 3Ge(OEt)_4_ | Sb_2_Te_3_ + 2(Me_3_Si)_3_Sb  → 4Sb + 3(Me_3_Si)_2_Te |
| Δ H | -733.53 kJ mol^-1^ | -218.99 kJ mol^-1^ |
| Δ G | -696.60 kJ mol^-1^ | -333.14 kJ mol^-1^ |

**Figures S1-S17**


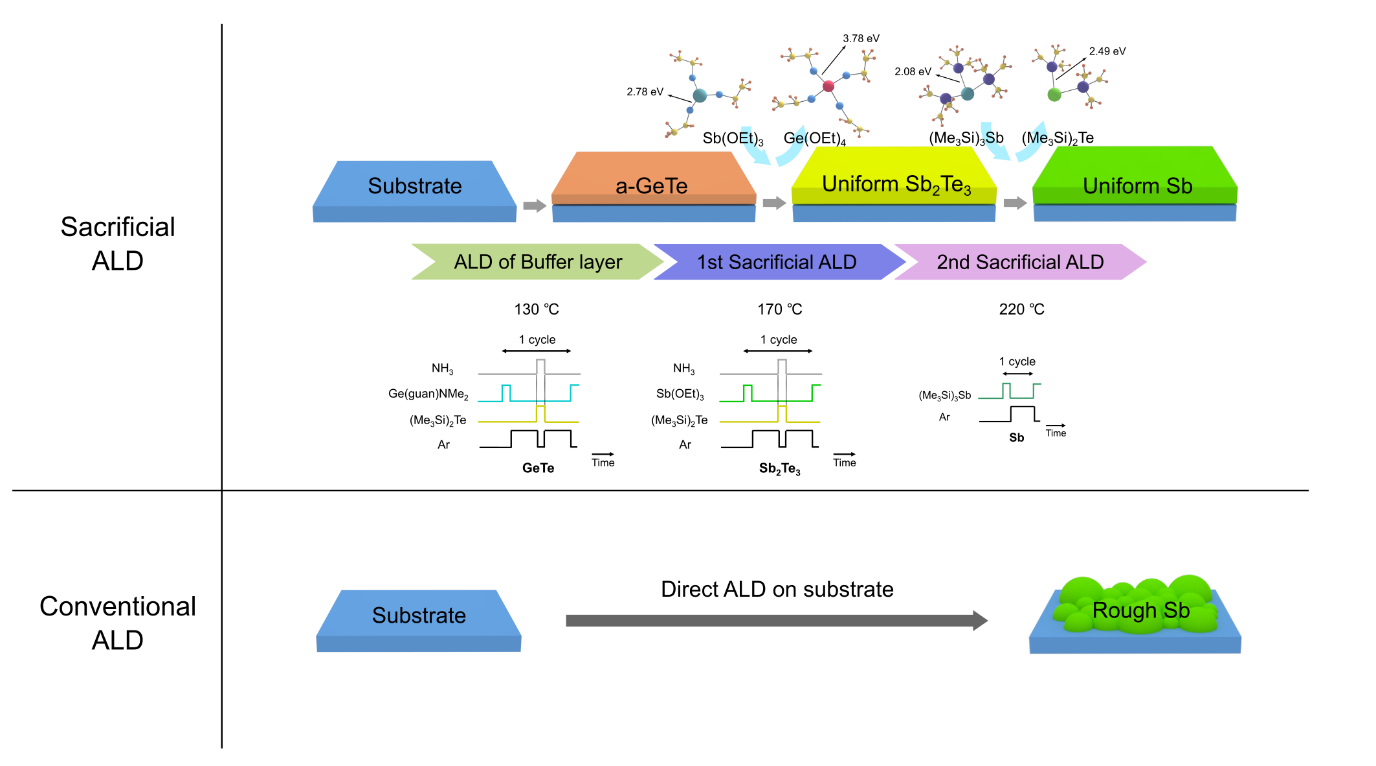


**Figure S1.** Detailed three-step s-ALD process for *c*-Sb film deposition. (upper row) s-ALD utilizes the difference in the bonding tendency between atoms and ligands to grow a film in a layer-by-layer mechanism, resulting in a uniform film. *a*-GeTe is deposited on the substrate and acts as a sacrificial buffer. Next, *c*-Sb_2_Te_3_ is deposited on *a*-GeTe, chemical substitution occurs, and results in a uniform *c*-Sb_2_Te_3_. Finally, (Me_3_Si)_3_Sb pulses are applied on *c*-Sb_2_Te_3_, chemical substitution occurs, and results in a uniform *c*-Sb. (lower row) Conventional ALD on the substrate could result in island growth with low surface coverage, leading to rough morphology.


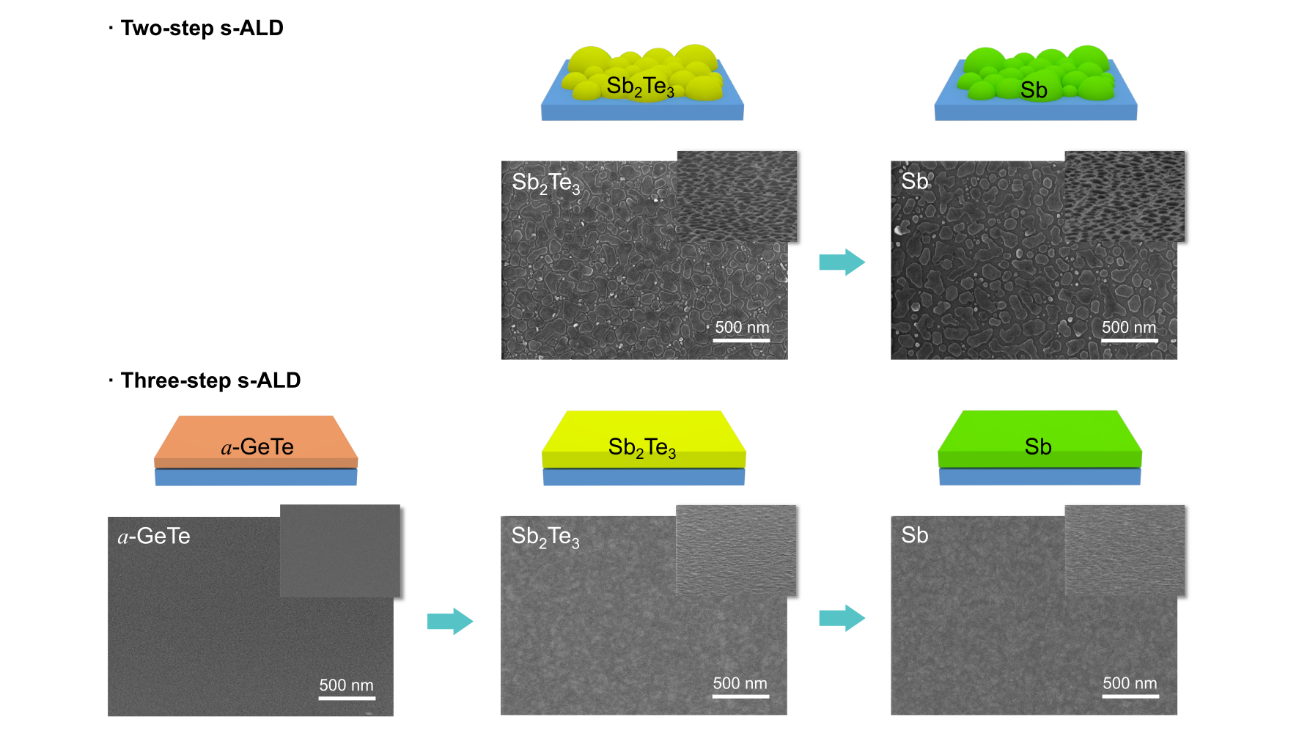


**Figure S2.** Comparison between two-step s-ALD and three-step s-ALD approaches for *c*-Sb film deposition. (upper row) Conventional ALD of *c*-Sb_2_Te_3_ on the substrate followed by conversion to *c*-Sb, resulting in a rough morphology. (lower row) s-ALD of *c*-Sb_2_Te_3_ incorporating *a*-GeTe buffer layer, followed by conversion to a monatomic *c*-Sb with uniform morphology and complete substrate coverage.


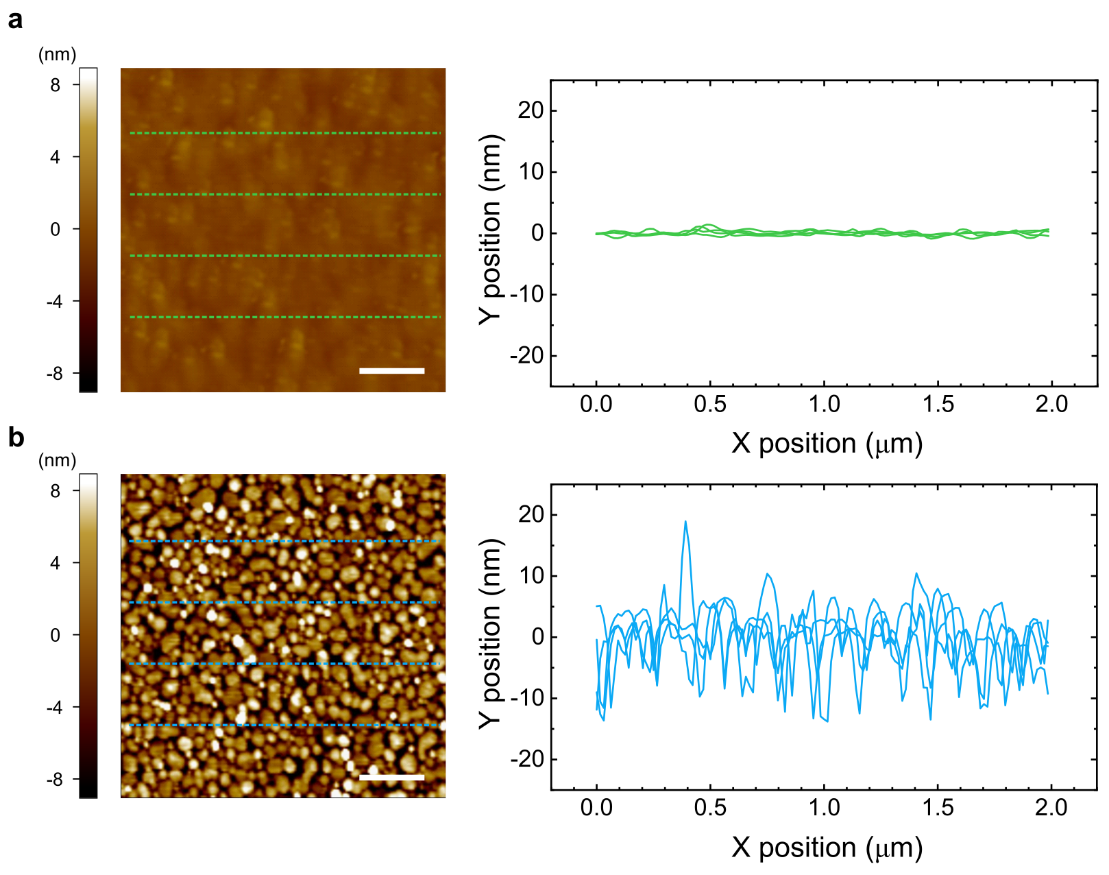


**Figure S3.** a,b) AFM line scans of the three-step s-ALD *c*-Sb film (a) and the island-type *c*-Sb film (b).


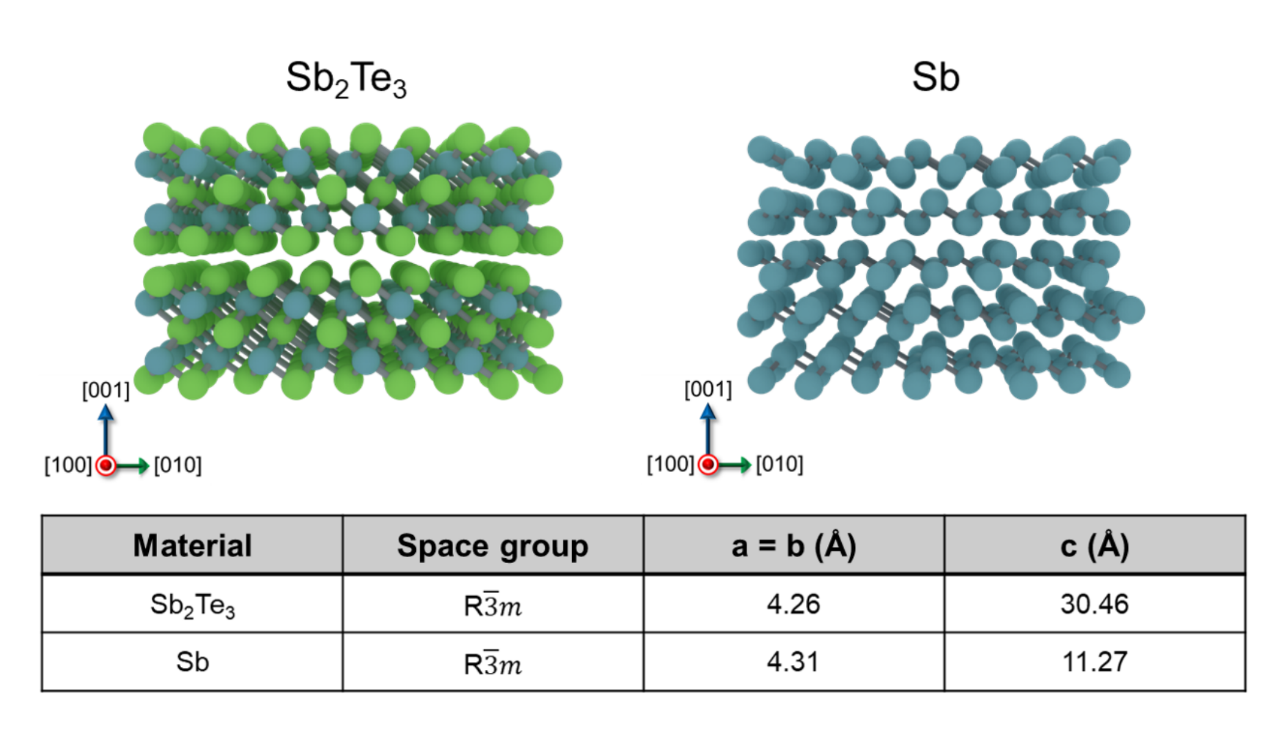


**Figure S4.** Crystallographic compatibility between Sb_2_Te_3_ and Sb. Both Sb_2_Te_3_ and Sb possess rhombohedral crystal structures and belong to the same space group (*R*$\overline{3}$*m*) with minimal in-plane lattice mismatch (~1%), enabling Sb_2_Te_3_ to provide an ideal template for the epitaxial growth of Sb.


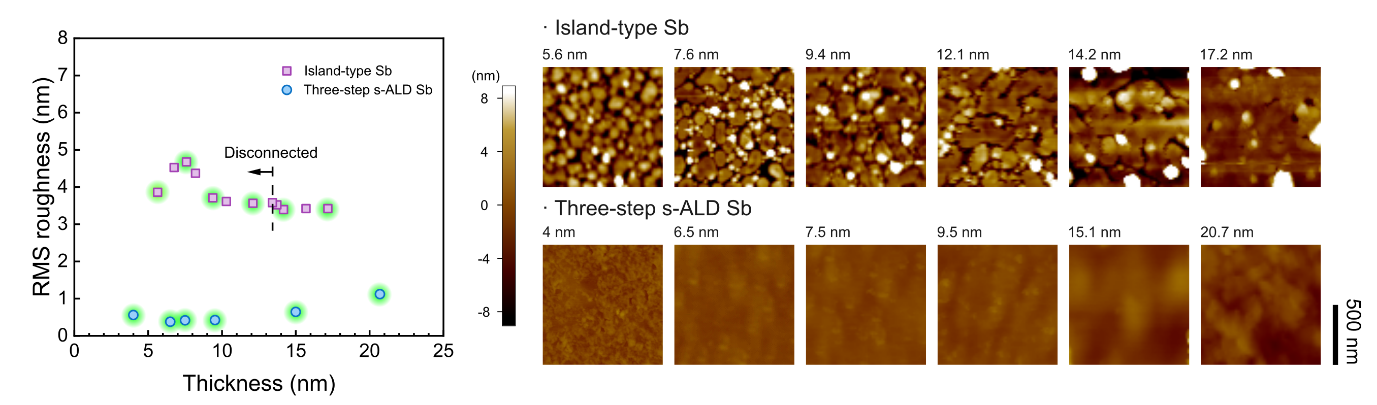


**Figure S5.** AFM images of island-type *c*-Sb and three-step s-ALD *c*-Sb at various thicknesses. Each image corresponds to the highlighted points.


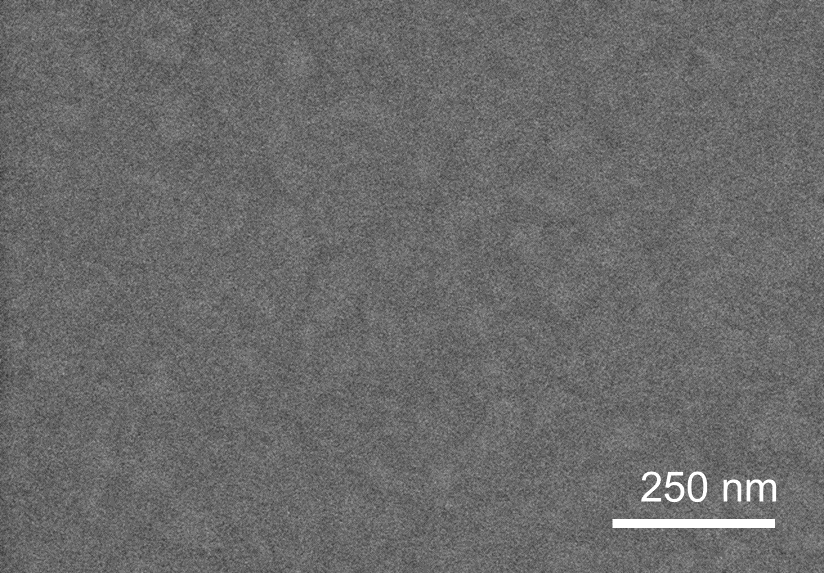


**Figure S6.** SEM image of an ultrathin (~4 nm) *c*-Sb film deposited on a SiO_2_ substrate with complete surface coverage despite the extreme thin film thickness.


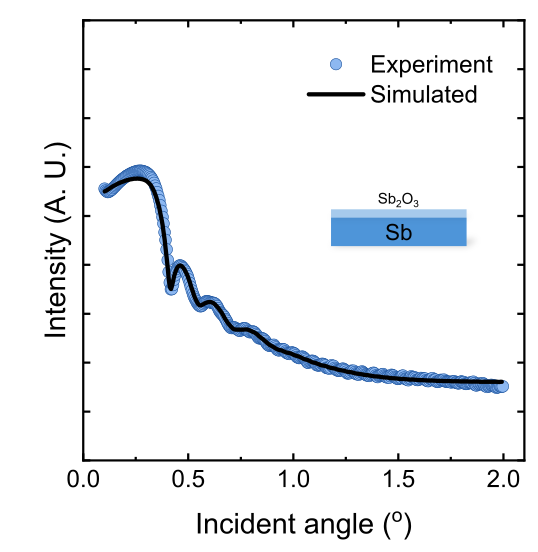


**Figure S7.** XRR result of the *c*-Sb film. The results were fitted with a native oxide layer, indicating a film bulk density of 6.52 g cm^-3^ for the Sb layer and 4.82 g cm^-3^ for the native oxide layer.


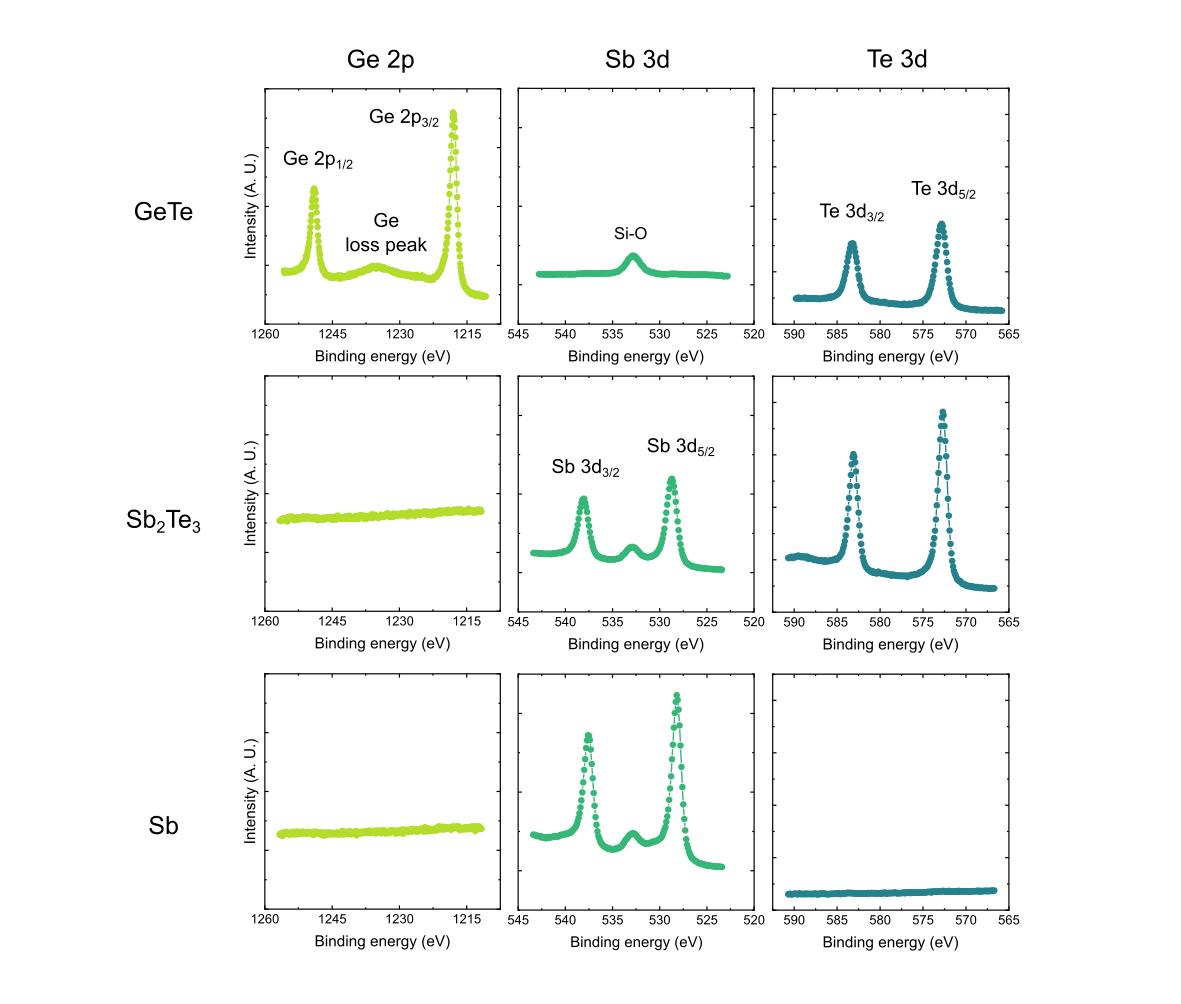


**Figure S8.** XPS spectra of *a*-GeTe, *c*-Sb_2_Te_3_, and *c*-Sb. (top) The *a*-GeTe film exhibits clear Ge 2p and Te 3d peaks; (middle) In the *c*-Sb_2_Te_3_ spectrum, Sb 3d and Te 3d peaks are present with negligible Ge signal; (bottom) The *c*-Sb film shows only Sb 3d peaks with no detectable Ge or Te signals. The Si-O peak shown in the Sb 3d region originates from the underlying SiO_2_ substrate.


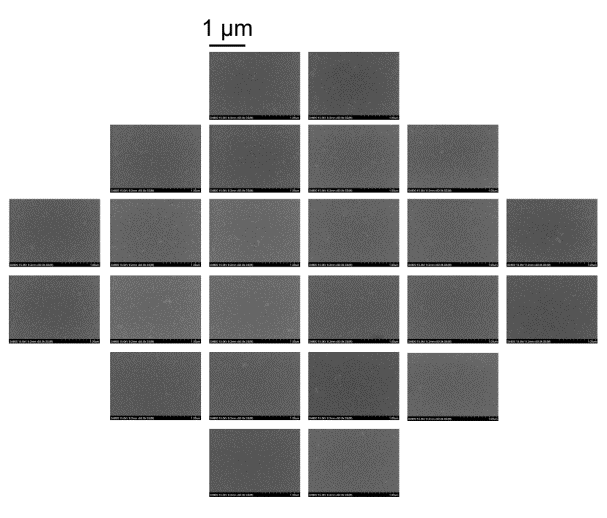


**Figure S9.** SEM images of s-ALD *c*-Sb acquired at 24 positions of the 4-inch wafer. Uniform morphology across the wafer is demonstrated.


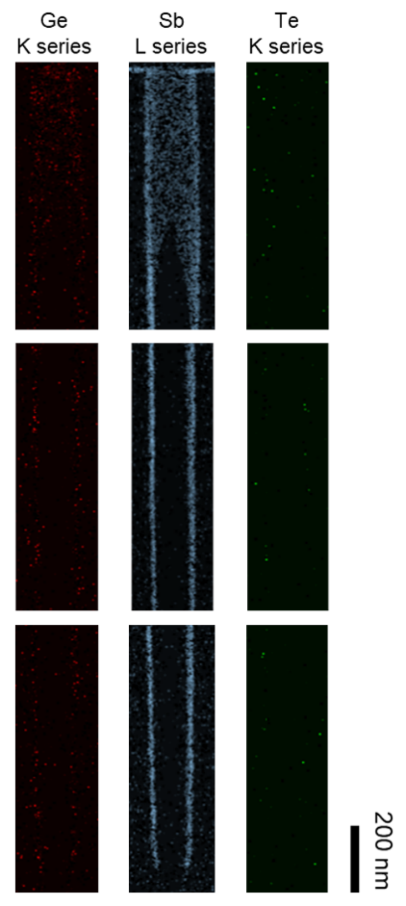


**Figure S10.** TEM-EDS images of the s-ALD *c*-Sb on a HAR hole structure. The Sb signal is visible while the Ge and Te signals are negligible. The slight Ge signal that appears on the upper section of the hole is due to the overlap of the Ge K line with the Pt L line from the Pt layer used during FIB sample preparation.


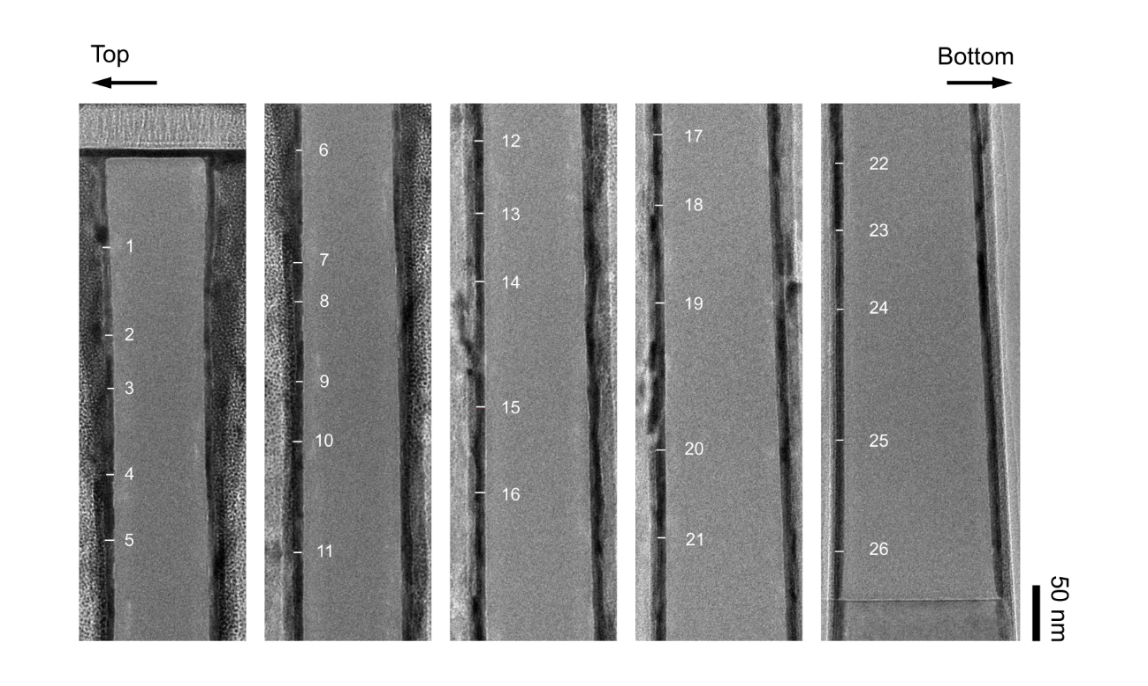


**Figure S11.** Magnified TEM images for thickness analysis of the *c*-Sb film deposited across the whole HAR structure. The points at which the thickness was measured are indicated by numbers.


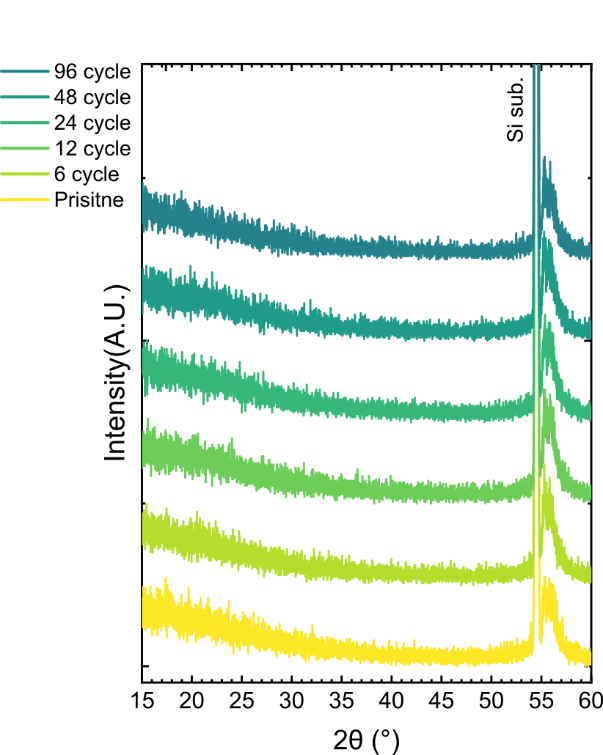


**Figure S12.** Glancing incidence X-ray diffractogram of the *c*-Sb_2_Te_3_/*c*-Sb films during the s-ALD process. No diffraction peaks corresponding to Sb_2_Te_3_ or Sb were observed, confirming that the (00*l*) alignment is consistently maintained throughout the entire substitution process.

**
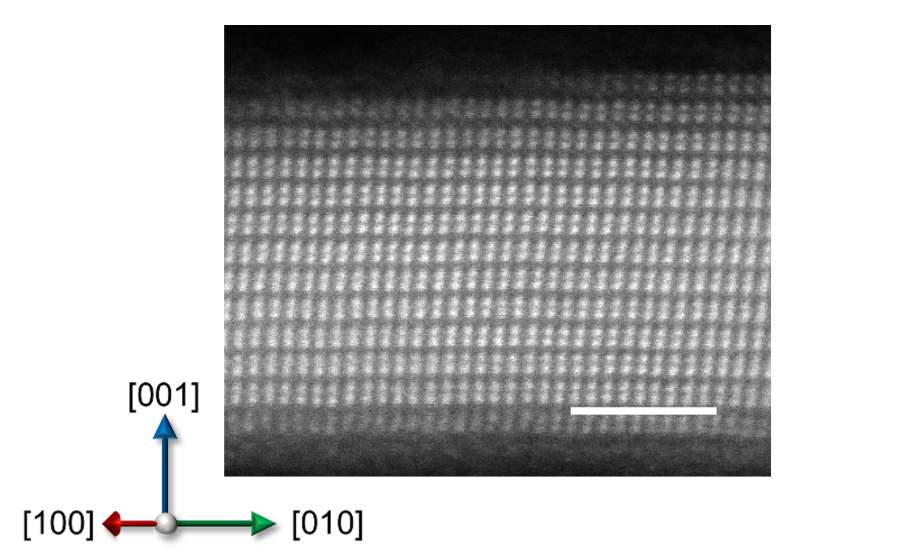
**

**Figure S13.** Cross-sectional STEM image of the *c*-Sb film viewed along the [-2 -1 0] zone axis. Scale bar: 2 nm.


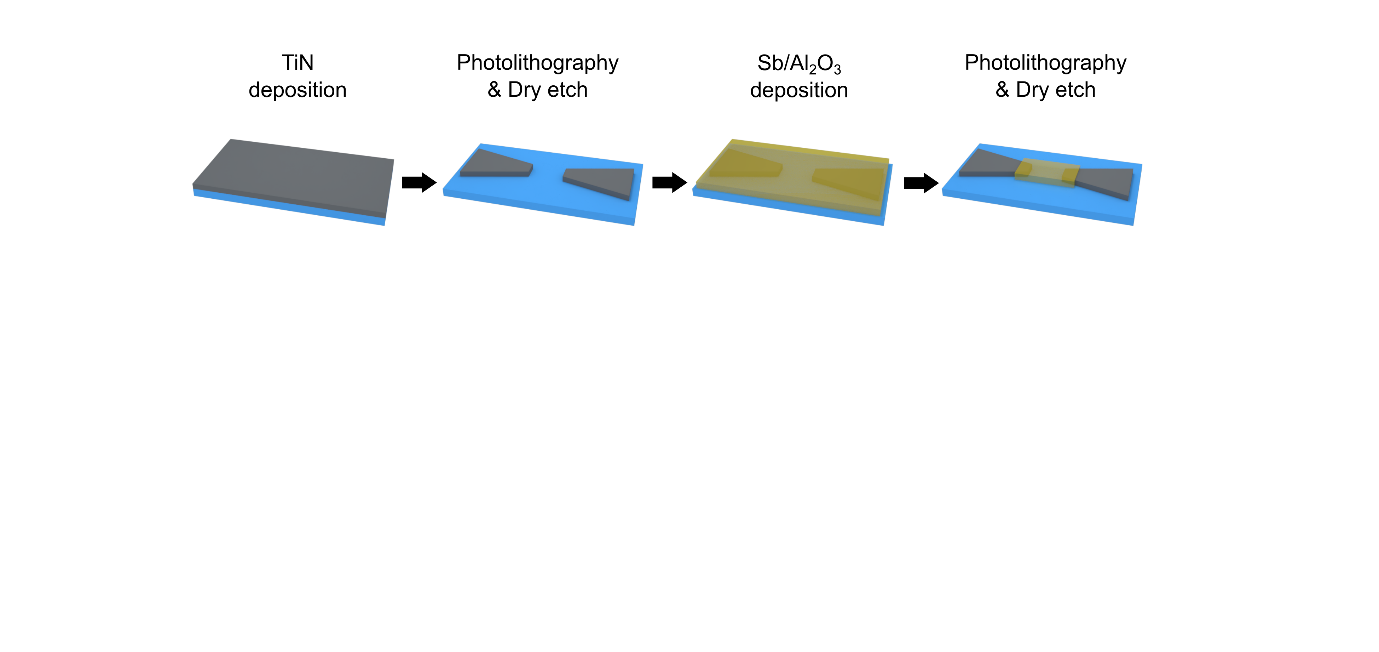


**Figure S14.** Process flow for fabricating monatomic Sb phase change memory devices. TiN bottom electrodes were formed by sputtering, photolithography and dry etching. s-ALD Sb active layer and Al_2_O_3_ capping layer are sequentially deposited, which were patterned using photolithography and dry etching.


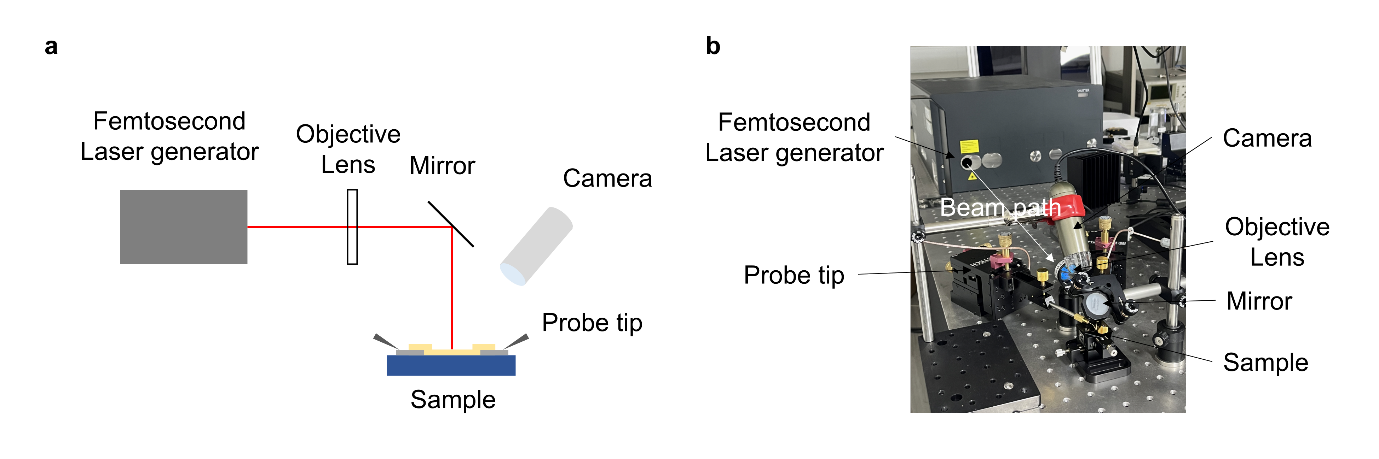


**Figure S15.** Lab-built PCM device testing system. a) Schematic illustration of the setup. b) Optical photograph of the experimental setup showing the main components. A femtosecond laser is focused through an objective lens onto the sample, while the probe tip measures the electrical resistance change of the sample.


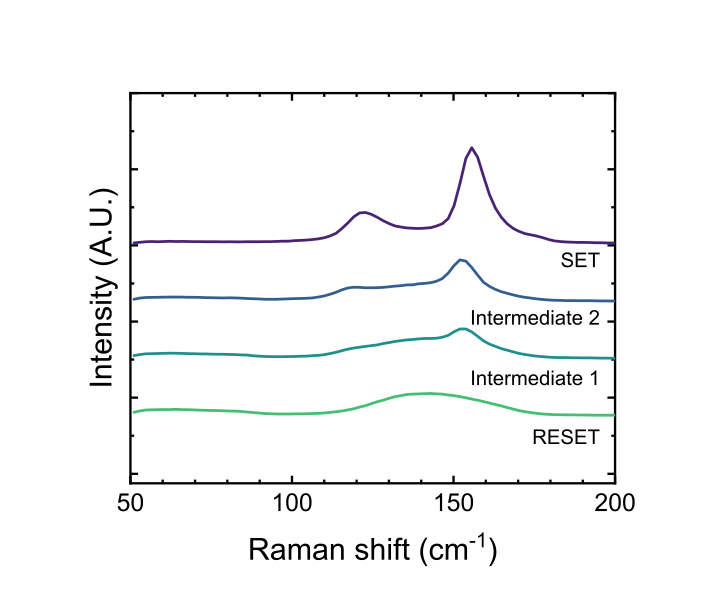


**Figure S16.** Raman spectra of the Sb films in the PCM device at SET, RESET, and intermediate resistance states.


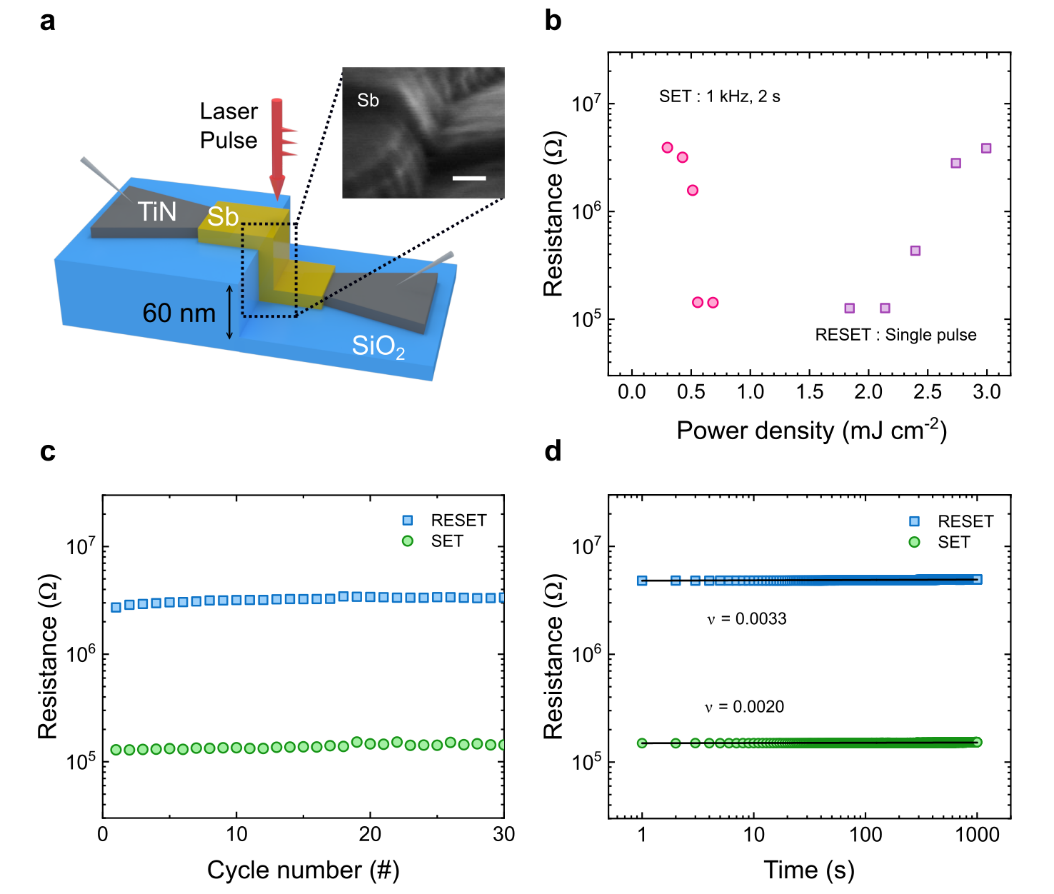


**Figure S17.** Ultrafast phase change memory properties of s-ALD Sb films in a vertical structure. a) Vertical PCM device configuration with SEM image. Scale bar: 50 nm. b) Resistance-fluence characteristics of the vertical Sb PCM device. c) Cycling performance of the vertical Sb PCM device. d) Resistance drift characteristics of the SET and RESET states.

**References**

[1] E. S. Park, C. Yoo, W. Kim, M. Ha, J. W. Jeon, Y. K. Lee, C. S. Hwang, *Chem. Mater.* **2019**, *31*, 8663.

[2] C. Yoo, W. Choi, S. Jeon, J. W. Jeon, B. Park, G. Jeon, I. H. Baek, C. S. Hwang, *Chem. Mater.* **2023**, *35*, 7311.

[3] Laidler Keith J., *Chemical Kinetics*, **1987**.

[4] J. Crank, *The Mathematics of Diffusion*, **1975**.
